# Proteomic Diversity in Bacteria: Insights and Implications for Bacterial Identification

**DOI:** 10.1016/j.mcpro.2025.100917

**Published:** 2025-01-27

**Authors:** Miriam Abele, Armin Soleymaniniya, Florian P. Bayer, Nina Lomp, Etienne Doll, Chen Meng, Klaus Neuhaus, Siegfried Scherer, Mareike Wenning, Nina Wantia, Bernhard Kuster, Mathias Wilhelm, Christina Ludwig

**Affiliations:** 1 Bavarian Center for Biomolecular Mass Spectrometry (BayBioMS), TUM School of Life Sciences, Technical University of Munich, Freising, Germany; 2 Chair of Proteomics and Bioanalytics, TUM School of Life Sciences, Technical University of Munich, Freising, Germany; 3 Computational Mass Spectrometry, TUM School of Life Sciences, Technical University of Munich, Freising, Germany; 4 Research Department Molecular Life Sciences, TUM School of Life Sciences, Freising, Germany; 5 Core Facility Microbiome, ZIEL Institute for Food & Health, Technical University of Munich, Freising, Germany; 6 Bavarian Health and Food Safety Authority, Unit for Food Microbiology and Hygiene, Oberschleißheim, Germany; 7 Institut für Medizinische Mikrobiologie, Immunologie und Hygiene, TUM School of Medicine and Health Department Preclinical Medicine, Technical University of Munich, Munich, Germany; 8 Munich Data Science Institute (MDSI), Technical University of Munich, Garching, Germany

**Keywords:** bacteria, microbes, proteomics, mass spectrometry, LC-MS/MS, diversity, bacterial identification, biotyping, proteotyping

## Abstract

Mass spectrometry–based proteomics has revolutionized bacterial identification and elucidated many molecular mechanisms underlying bacterial growth, community formation, and drug resistance. However, most research has been focused on a few model bacteria, overlooking bacterial diversity. In this study, we present the most extensive bacterial proteomic resource to date, covering 303 species, 119 genera, and five phyla with over 636,000 unique expressed proteins, confirming the existence of over 38,700 hypothetical proteins. Accessible *via* the public resource ProteomicsDB, this dataset enables quantitative exploration of proteins within and across species. Additionally, we developed MS2Bac, a bacterial identification algorithm that queries NCBI's bacterial proteome space in two iterations. MS2Bac achieved over 99% species-level and 89% strain-level accuracy, surpassing methods like MALDI-TOF and FTIR, as demonstrated with food-derived bacterial isolates. MS2Bac also effectively identified bacteria in clinical samples, highlighting the potential of MS-based proteomics as a routine diagnostic tool.

## Highlights

• Bacterial proteomic resource covering 303 species, 119 genera, and 636,000 proteins.
• All data are available through ProteomicsDB.
• MS2Bac - a novel bacterial identification algorithm.
• MS2Bac achieves >99% species-level and >89% strain-level accuracy.
• MS2Bac accurately identifies bacteria in food-derived and clinical samples.

## Graphical Abstract

**Figure:**
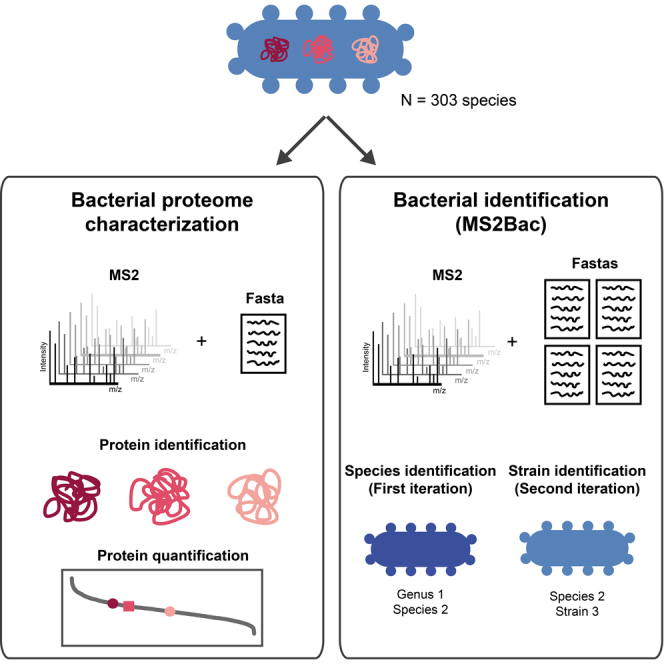

## In Brief

Mass spectrometry–based proteomics has advanced bacterial identification and revealed key molecular mechanisms. However, most studies focus on few model bacteria, overlooking diversity. Here, we present the largest bacterial proteomic resource, covering 303 species and over 636,000 unique proteins, available *via* ProteomicsDB. We also developed MS2Bac, a bacterial identification algorithm achieving over 99% species-level and 89% strain-level accuracy. MS2Bac outperforms MALDI-TOF and FTIR and effectively identifies bacteria in clinical samples, showcasing its potential for routine diagnostics.

Large (meta-)genomic projects aim to catalog the global phylogenetic diversity of the bacterial domain and describe the composition of microbiological ecosystems (1, 2). In stark contrast to the 13,000 species' genomes deposited in the National Center for Biotechnology Information sequence database (NCBI, status 2023-02-17) (3), few bacterial species have been experimentally characterized on a proteome-wide scale to date. Knowledge about the existence of a protein, its expression level, posttranslational modifications, localization, structure, and the overall protein composition of a bacterium is crucial for unraveling the mechanisms underlying microbial cell function (4, 5). The two most diverse mass spectrometry (MS)-based proteomics studies published to date characterized the proteomes of 49 and 23 bacterial species and described around 65,000 and 45,000 proteins, respectively (6, 7). Ideally, such qualitative and quantitative proteome information is freely accessible to the community through public resources such as ProteomicsDB (https://www.proteomicsdb.org/; (8)), which offers a graphical user interface for multiomics integration.

One critical question in microbiology concerns bacterial identification. Fast, reliable, and accurate identification algorithms are essential for basic research, treatment decisions in the health sector, epidemiological intervention, and economic success, for example, in the food industry. Historically, biochemical, serological, and chemotaxonomical approaches were applied. Later, these were replaced by more rapid and efficient technologies like 16S rRNA gene sequencing and matrix-assisted laser desorption ionization time-of-flight mass spectrometry (MALDI-TOF). Whole genome sequencing and Fourier-transformed infrared spectroscopy (FTIR) are less commonly applied technologies. Whole genome sequencing has long turnaround times, and the other methods require pure cultures and have limited taxonomic resolution and accuracy, especially for closely related taxa (9). Identification approaches employing bottom-up proteomics as an analytical method have been proposed due to their capability to provide protein-level information, for example, on antibiotic resistance markers (10, 11), as well as their potential for (sub-)species-level identifications (12). These algorithms can be divided into four categories: (i) identification on precursor-level (13), (ii) identification of single or multiple diagnostic peptides that are unique to one species (14, 15, 16, 17), (iii) identification on tryptic peptide signatures (18), and (iv) identification on all identified tryptic peptides at 1% false-discovery rate (FDR) (19, 20, 21). However, MS-based proteomics has so far been applied to datasets with limited taxonomic depth (*e.g.*, five *Burkholderia thailandensis* strains (13)) and breadth (*e.g.*, 15 different species (18)). A comprehensive validation dataset is missing to estimate the accuracy of bottom-up proteomics for bacterial (sub-)species identification. Furthermore, new species are discovered rapidly, and reference databases are continuously growing. The consequence is that peptide identifications at 1% FDR decrease due to the search space explosion.

In this study, we applied MS-based bottom-up proteomics to quantitatively characterize the proteomes of 303 diverse bacterial species, providing evidence for over 625,000 unique expressed proteins. The complete dataset has been implemented into the public resource ProteomicsDB, which offers a graphical user interface to explore the quantitative data within one organism and across species. Further, this dataset was used to develop a new bacterial identification algorithm called MS2Bac which uses a two-iteration approach to identify bacteria, first, on species- and, second, on strain-level. Instead of a 1% FDR, MS2Bac assigns all peptide-spectrum matches to reference bacteria. MS2Bac then reports the species (or strain) with the highest counts as the identified organism. We further complemented this dataset with strain-specific datasets and a reproducibility study to demonstrate the merits of bacterial identification with bottom-up proteomics. Accuracies of >99% and >89% can be achieved at the species- and strain-levels, respectively. Finally, we show that proteomics-based identification provided more accurate results than MALDI-TOF or FTIR and applied the method to 570 clinical isolates.

## Experimental Procedures

### Dataset Overview

An overview of all experiments and their respective sample preparation, data acquisition, and data analysis details is listed in Supplemental Table S1. Briefly, we acquired eight datasets: (i) 14 samples per day (SPD) diversity dataset (proteome profiling of 318 strains from 303 species, 90-min gradient), (ii) 38 SPD diversity dataset, (iii) 80 SPD diversity dataset, (iv) reproducibility dataset, (v) *Pseudomonas* spp. dataset (94 *Pseudomonas* spp. strains), (vi) *Bacillus* spp. dataset (28 *Bacillus cereus* s.l. strains), (vii) food dataset (60 dairy product isolates), and (viii) clinical dataset (570 clinical isolates). Species for the diversity dataset were selected based on (i) taxonomic diversity (according to NCBI), (ii) availability at German Collection of Microorganisms and Cell Cultures GmbH (DSMZ) or the American Type Culture Collection (ATCC), (iii) assembled genome sequence on strain-level at NCBI, (iv) preference for type strains, and (v) biological safety level below or equal to biosafety level 2 according to the German classification, as stipulated in TRBA 466 (22).

### Experimental Design and Statistical Rationale

All samples were measured in singlicates, except for samples in dataset (iv). Here, each condition was measured in biological triplicates to estimate the workflow's reproducibility. A *Pseudomonas aeruginosa* quality control (QC) sample was included in most of the datasets. Bacterial cells from several cultivation plates were pooled, and cell pellet aliquots were stored at −80 °C. This QC sample was lysed, digested, desalted, and measured with samples from the diversity dataset. Information about which samples and QCs were lysed and processed together is provided in Supplemental Table S2. In total, 23 *P. aeruginosa* QCs were included in datasets (i) and (ii) to track technical variance throughout the sample preparation and data acquisition process. In dataset (iii), 22 QCs were included. Samples from dataset (iv) were all processed on the same day and measured sequentially on the mass spectrometer within 3 days. Datasets (v) and (vii) included two QC samples each. Statistical methods are explained in more detail in the “Bioinformatic analysis” paragraph.

### Bacterial Cell Culture

All cultures for the diversity datasets were obtained from strains stored in the Weihenstephan Microbial Strain Collection (https://ccinfo.wdcm.org/details?regnum=1163). All strains had originally been purchased from the German Collection of Microorganisms and Cell Cultures GmbH (DSMZ) or the ATCC (Supplemental Table S2). Glycerol stocks were stored at −80 °C. To activate cultures, bacteria were passaged at least three times. Bacteria were cultured on their respective agar plate types, temperatures, and oxygen conditions in a confluent cell lawn (Supplemental Table S2). Anaerobic conditions were achieved by culturing bacteria in a plastic bag, applying a vacuum, and using Anaerocult A mini (Merck KGaA). Microaerophilic bacteria were grown the same way but without a vacuum at first. CVUA strains in dataset (vi) were provided by the “Chemischen und Veterinäruntersuchungsamt Stuttgart.” De-identified left-over isolates from dairy products were obtained from the routine diagnostic laboratory for food-derived samples at the Chair of Microbial Ecology, School of Life Sciences, Technical University of Munich. De-identified left-over clinical isolates were obtained from the routine diagnostic laboratory of the Institute for Medical Microbiology, School of Medicine and Health, Technical University of Munich, observing the appropriate ethics approval process of the Technical University of Munich (2024-103-NM-BA). Concerning both, all isolates were sampled directly from agar plates grown under conditions determined by standard operation procedures from the routine laboratories. Thermophilic bacteria from the dairy routine dataset were grown at 55 °C. We cannot rule out that some clinical isolates were grown on antibiotic sensitivity–testing agar plates. Biomass subjected to a tryptic digest for mass-spectrometric measurements was directly sampled from agar plates.

### Cell Lysis

Bacteria were lysed with 100% TFA according to the SPEED protocol (23) with slight adaptations (7). Shortly, 50 μl of 100% TFA was added to the cell pellet, heated for 5 min at 55 °C, and neutralized with 450 μl 2 M Tris (pH not adjusted). The pH after neutralization was around 8.2.

### In Solution Protein Digest

Supplemental Table S3 provides a detailed summary of sample preparation strategies used for the individual experiments.

Protein concentrations were determined with the Bradford assay (Thermo Fisher Scientific) according to manufacturer's instructions. For deep proteome profiling of 303 species and the two genus-specific datasets (datasets (i), (v), (vi)), 50 μg protein was reduced and alkylated (9 mM tris(2-carboxyethyl)phosphine and 40 mM chloroacetamide for 5 min at 95 °C) in one step. In 19 samples, the input amount was less than 50 μg because insufficient biological material was available (Supplemental Table S2). Samples were diluted with deionized water to a final concentration of 1 M Tris and 5% TFA. Trypsin (Roche) was added in a trypsin:protein ratio of 1:50 to all samples, for example, 1 μg trypsin for 50 μg protein. Samples were incubated overnight at 30 °C and 450 rpm on a ThermoMixer C (Eppendorf SE). The next day, all samples were acidified to a final concentration of 3% formic acid (FA). In the case of samples from datasets (ii)–(iv), (vii), and (viii), 20 μg protein was reduced and alkylated under the same conditions as described before. Trypsin was added in trypsin:protein ratio of 1:20 to all samples and incubated for 2 h at 30 °C and 450 rpm.

### C18 Peptide Cleanup

All digested samples were desalted. Briefly, self-packed desalting tips (24) were prepared in-house, consisting of three Empore C18 (3M) disks for 20 μg protein input or five for 50 μg protein input. Tips were primed with 100% acetonitrile (ACN), then 40% ACN/0.1% FA, and finally equilibrated with 0.1% FA. Peptides were loaded, washed with 0.1% FA, and eluted twice with 40% ACN/0.1% FA. Samples were lyophilized before the dried peptides were stored at −80 °C.

### LC-ESI-MS/MS Measurements

Supplemental Table S4 lists a detailed summary of data-acquisition strategies for the individual experiments.

#### Nanoflow LC–Fusion Lumos

Peptides were analyzed on an UltiMate 3000 RSLCnano (Thermo Fisher Scientific) coupled to an Orbitrap Fusion Lumos Tripbrid mass spectrometer (Thermo Fisher Scientific). For the *Pseudomonas* spp. and *Bacillus* spp. datasets (v) and (vi), around 500 ng of peptides spiked with 50 fmol retention time standardization kit peptides (Procal (25); JPT Peptide Technologies GmbH) were applied onto a trap column (ReproSil-pur C18-AQ, 5 μm, Dr Maisch HPLC GmbH (Ammerbuch-Entringen), 20 mm × 75 μm, self-packed) for 10 min and at a flow rate of 5 μl/min in HPLC grade water with 0.1% (v/v) FA. Peptides were separated on an analytical column (3 μm C18 resin, ReproSil Gold Dr Maisch HPLC GmbH (Ammerbuch-Entringen, 450 mm × 75 μm, self-packed) over a 30 min linear gradient ranging from 4% to 34% of solvent B (0.1% (v/v) FA, 5% (v/v) dimethylsulfoxide (DMSO) in ACN) at 300 nl/min flow rate. The nano-LC solvent A was 0.1% (v/v) FA and 5% (v/v) DMSO in HPLC-grade water.

The mass spectrometer was operated in data-dependent acquisition (DDA) and positive ionization mode. MS1 full scans (360–1300 m/z) were acquired with a resolution of 60,000, a normalized automatic gain control target value of 400,000, and a maximum injection time of 50 ms. Peptide precursor selection for fragmentation was carried out using a cycle time of 2 s. Only precursors with charge states from two to six were selected, and dynamic exclusion of 25 s was enabled. Peptide fragmentation was performed using higher energy collision-induced dissociation and normalized collision energy of 30%. The precursor isolation window width of the quadrupole was set to 0.7 m/z. MS2 spectra were acquired with a resolution of 15,000, a fixed first mass of 100 m/z, a normalized automatic gain control target value of 100,000, and a maximum injection time of 25 ms.

#### Microflow LC–Orbitrap Exploris 480

Peptides were analyzed on a Vanquish Neo UHPLC (microflow configuration; Thermo Fisher Scientific) coupled to an Orbitrap Exploris 480 mass spectrometer (Thermo Fisher Scientific). For proteome profiling, around 25 μg of peptides were applied onto a commercially available Acclaim PepMap 100 C18 column (2 μm particle size, 1 mm ID × 150 mm, 100 Å pore size; Thermo Fisher Scientific) and separated on a stepped gradient from 3% to 31% solvent B (0.1% FA, 3% DMSO in ACN) in solvent A (0.1% FA, 3% DMSO in HPLC grade water) over 90 min. A flow rate of 50 μl/min was applied. The mass spectrometer was operated in DDA and positive ionization mode. MS1 full scans (360–1300 m/z) were acquired with a resolution of 60,000, a normalized automatic gain control target value of 100%, and a maximum injection time of 50 ms. Peptide precursor selection for fragmentation was carried out using a cycle time of 1.5 s. Only precursors with charge states from two to six were selected, and dynamic exclusion of 35 s was enabled. Peptide fragmentation was performed using higher energy collision-induced dissociation and normalized collision energy of 28%. The precursor isolation window width of the quadrupole was set to 1.1 m/z. MS2 spectra were acquired with a resolution of 15,000, a fixed first mass of 100 m/z, a normalized automatic gain control target value of 100%, and a maximum injection time of 40 ms.

For datasets (ii) and (iii), the linear gradient was shortened to 30 min and 10 min, respectively. The LC gradient was changed to a linear gradient from 3% to 28% solvent B, and only 10 μg peptides were applied. All other parameters remained the same. Routine diagnostic isolates (dairy product and clinical datasets (vii) and (viii)) were measured with the 10-min gradient method.

### Identification and Quantification of Peptides and Proteins

For all proteome profiling analyses, MaxQuant v1.6.3.4 with its built-in search engine Andromeda (26, 27) was used for peptide identification and quantification. MS2 spectra were searched against their respective proteome stored as an amino acid fasta file (Supplemental Table S2). Proteomes were obtained from NCBI (3) on Nov 27th and 28th, 2023 and supplemented with common contaminants (built-in option in MaxQuant). Trypsin/P was specified as the proteolytic enzyme. Precursor tolerance was set to 4.5 ppm, and fragment ion tolerance to 20 ppm. The minimal peptide length was defined as seven amino acids; the “match-between-run” function was disabled. Carbamidomethylated cysteine was set as a fixed modification, and methionine oxidation and N-terminal protein acetylation were defined as variable modifications. The FDR was set to 1, meaning 100%, to allow for a subsequent peptide-spectrum match (PSM) rescoring *via* Oktoberfest (28) that uses Prosit (29, 30) and Percolator (31), implemented in ProteomicsDB (intensity: HCD, model from 2020; iRT: model from 2019; (8)).

#### Analysis on ProteomicsDB

On ProteomicsDB, we define an organism as an entity that has a unique taxcode and, thus, protein space and is at the lowest phylogenetic level. In the context of this project, each strain is a distinct organism. Following rescoring, PSMs and peptides were filtered to achieve a 1% FDR, calculated at the sample-, experiment-, and the organism-level. ProteomicsDB used only protein- or gene-specific peptides with a q-value lower or equal to 0.01 for protein identification and quantification. Protein- and gene-level FDR estimates were then computed using the picked target-decoy approach (32) and the results were filtered to achieve a 1% FDR at the sample-, experiment-, and the organism-level. Controlling the FDR at the organism level (*i.e.*, across all experiments and samples for a given organism) prevents error accumulation across analyses. Since each organism is searched against its own distinct protein database, there is neither a need nor a practical way to control FDR at a higher (across organisms) level. All peptide sequences, proteins, intensities, mass-spectra, and corresponding information are available in ProteomicsDB. Intensity-based absolute quantification (iBAQ) (33) was used as a protein abundance estimator and calculated as previously described. iBAQ intensities were normalized by dividing each protein's abundance by the total sum of iBAQ values within the respective sample, producing relative iBAQ values. These relative iBAQs are then log_10_-transformed and further shifted by 10 units to maintain positive values. For cross-organism analysis, where comparable abundance distributions are required, ProteomicsDB normalized the log_10_-transformed iBAQ values to standard scale (μ = 0, σ = 1) and right-shifted by five units into positive numerical space (z-scored iBAQ).

#### Offline Analysis

The coefficient of variance was calculated on median-centered iBAQ values. Unless otherwise stated, we used z-scored iBAQ values for this paper (equal to the cross-organism analysis on ProteomicsDB, see above).

For the reproducibility experiment (dataset (iv)), we performed a 1% FDR MaxQuant search to describe the qualitative and quantitative differences between the samples. We used the peptide.txt output file and analyzed the data in Perseus v2.0.6.0 (34). First, “potential contaminants” and “reversed” (=decoys) and all peptides that had missed cleavages were removed. Second, peptide LFQ intensities (35) were log_2_ transformed. Third, we used the visualization option “Hawaii plot” with default parameters, exported the results table, and plotted volcano plots in Python with the libraries matplotlib (36) and seaborn (37). We highlighted peptides with a *p*-value >0.01 and a fold change >2.

Raw files from clinical isolates were analyzed with MaxQuant v1.6.3.4 to detect antibiotic resistance markers at 1% FDR. MS2 spectra were searched against the identified strain from MS2Bac appended with the protein sequences from the Comprehensive Antibiotic Resistance Database (file: protein_fasta_protein_homolog_model.fasta, entries: 4775) (38). We removed proteins that were “reversed” (=decoys), “only identified by site,” “potential contaminant,” had less than two peptides per protein or an iBAQ = 0.

### 16S rRNA Gene Sequencing

We sequenced the 16S rRNA gene from 60 dairy product isolates to determine the bacterial taxonomy. One inoculation loop of bacteria was mixed with 200 μl DNAse/RNAse free water for cell lysis. Mechanical cell lysis was performed using bead beating (Fast Prep-24 from MP Biomedicals; 0.1 mm zirconia beads; two cycles of 45 s at 6.5 m/s). Samples were incubated at 95 °C and 600 rpm in a ThermoMixer C (Eppendorf SE). Samples were centrifuged at 10,000 rcf at room temperature. The concentration of the supernatant was determined with a Nanodrop (Eppendorf SE) and diluted to 20 ng/μl with PCR-grade water. For PCR, 15 μl master mix (10 μl 5x GC buffer, 1 μl 10 mM dNTP, 1 μl 10 μM forward primer 1 (5′-AGA GTT TGA TCC TGG CTC AG-3′), 1 μl 10 μM forward primer 2 (5′-AGA GTT TGA TCA TGG CTC AG-3′), 1 μl 10 μM reverse primer (5′-ACG GTT ACC TTG TTA CGA CTT-3′), 31.6 μl DNAse/RNAse free water, and 0.4 μl *Taq* polymerase) was mixed with 5 μl DNA. In total, 35 PCR cycles (94 °C, 5 min (only in the first cycle) –94 °C, 1 min –43 °C, 1 min, 72 °C, 2 min –72 °C, 7 min, and finally 10 °C on hold) were performed. Subsequently, 0.2 μl PCR product, 2 μl reverse primer, and 14.2 μl PCR grade water were mixed and sent for sequencing. Sequencing results were searched against three different databases (EZBioCloud, https://www.ezbiocloud.net/ (39); NCBI “16S ribosomal RNA sequences” and NCBI “Nucleotide collection (nr/nt)”, https://blast.ncbi.nlm.nih.gov/Blast.cgi; (40)). Identification criteria included >98% sequence similarity for species differentiation and a minimum of two out of three equal matches in the three database searches. In three cases, no conclusive 16S rRNA gene sequencing result was obtained, and in two more cases, only the genus was identified.

### FTIR Spectroscopy

Bacteria were grown under the same conditions as during reference database creation: Lactic acid bacteria were incubated anaerobically for 24 h ± 0.5 h at 34 °C on APT agar. Thermophilic bacilli were incubated aerobically for 24 h ± 0.5 h at 55 °C on TS agar. Mesophilic bacilli were incubated aerobically for 24 h ± 0.5 h at 25 °C on tryptic soy agar. One inoculation loop of bacteria was homogenized in 100 μl autoclaved, deionized water. Twenty microliters of each sample were transferred onto a 96-well zinc selenide sample carrier and dried for around 30 min at 40 °C. Samples were measured with a Tensor 27 spectrometer coupled to an HTS-XT high-throughput instrument (both Bruker Optics), according to Wenning *et al*. (9). Spectra were searched against in-house databases (*Pseudomonas*, spore-forming bacteria at 55 °C, spore-forming bacteria at 25 °C, Enterobacteriaceae, lactic acid, or G+ nonspore-forming bacteria). Identification criteria were the same as previously described (9).

### MALDI-TOF Mass Spectrometry

Bacteria were picked from agar plates with a sterile toothpick, spotted on a MALDI steel target (Bruker Daltonics), and dried. Afterward, 1 μl 70% FA solution and 1 μl alpha-cyano-4-hydroxycinnamic acid (solved in 250 μl 50% acetonitrile, 47.5% water, and 2.5% TFA; kept in the dark) were added to each spot. Between these two steps, samples were dried. Mass spectra were acquired by a Microflex LT MALDI-TOF MS (Bruker Daltonics GmbH & Co KG). The analysis software was the MALDI Biotyper 3.0 Realtime classification (Bruker Daltonics) following (41). Genus identification was possible for hits with scores ≥1.7, and species identification was possible for hits ≥2.0. The reference database contained 7279 entries.

### Bioinformatic Analysis

We used NCBI's Taxonomy browser to create common trees (42). We visualized them with the Python package pyCirclize (Y. Shimoyama, Github: https://github.com/moshi4/pyCirclize) and iTOL (43).

Ortholog mapping was performed using OrthoFinder v2.5.5 (44) with default parameters for fasta files listed in Supplemental Table S5. Orthogroups (OGs) were inferred on the reference proteomes retrieved from NCBI, as previously described. OGs can entail paralogs (proteins with a high sequence similarity from the same strain) and orthologs (proteins with a high sequence similarity from a different strain). An OG is always composed of at least two protein entries. A table with all OGs is available as Supplemental Table S6.

We computed taxonomic conservation classes (domain – D, phylum – P, class – C, family – F, genus – G) to reflect the conservation degree of an orthogroup based on the fasta files listed in Supplemental Table S5. First, a binary, taxon-specific conservation array (TSCA) was computed using ‘1s’ (OG is expected to be present in the proteome) and ‘0s’ (OG is expected to be absent in the proteome). For example, a domain-specific TSCA consisted of only “1s,” while a Bacillota-specific TSCA contained “1s” for all Bacillota species and “0” for all other species. Second, we defined OG-specific arrays, in which a ‘1’ indicated that at least one protein from the respective OG was found in the reference proteome (= NCBI fasta files). In contrast, ‘0s’ indicated no protein exists for this OG in the respective species. Finally, similarities between TSCAs and OG-specific arrays were compared using the hamming distance, and every OG was classified into the taxonomic conservation class with the lowest hamming distance.

In the case of “expression *versus* degree of conservation” calculations, we filtered for a class-specific hamming distance reflecting the 75% quantile. We additionally filtered for the median z-scored iBAQ value of paralogs. Then, we used the species with the maximum value for the overall orthogroup expression level.

Protein sequences were annotated using the InterProScan pipeline v5.66-98.0 (45) using the default parameters (except for the databases that were set to SMART, Pfam, FunFam, Hamap, and NCBIfam) and PANNZER2 (46) (accessed in January 2024) with default parameters. The retrieved gene ontology (GO) terms were aggregated for each protein ID, and the obsolete terms were removed based on the gene ontology database (release 17 January 2024) (46, 47, 48).

GO term enrichment analysis was performed using the GOATOOLS v1.3.11 package (49) in Python v3.10.11. Fisher's exact test was defined as the statistical measure. The Benjamini-Hochberg FDR approach was used for multiple hypotheses testing with a significance threshold of 0.05. GO term enrichment analysis was conducted separately for the top 10 expressed proteins in each strain, using the identified proteins within the respective strain as the background. The output with significant hits is summarized in Supplemental Table S7.

The current knowledge of protein existence was determined based on the Uniprot/SwissProt database (50), where we downloaded all proteins with the taxonomy reference “taxid:2” (=Bacteria) and “evidence at protein level” (n = 23,933). We then mapped identified peptides from the diversity dataset to the *in silico* tryptic digests (up to two missed cleavages and an amino acid length 7–30) of these 23,933 proteins. If a peptide was not found in the Uniprot-derived peptide list, the inferred protein was annotated as novel in this manuscript.

Pairwise Jaccard similarity indices were computed to compare tryptic peptidomes between species or strains. We used one reference strain per species for this analysis and included only bacteria from the phyla Pseudomonadota, Bacillota, and Actinomycetota. Selection criteria for these representative strains were as follows: (i) NCBI average nucleotide identity (ANI) status is “OK,” (ii) the species name must be known, (iii) the species taxon contains at least one “representative genome” or a “reference genome.” Due to the high number of strain genomes deposited in NCBI, we limited the strain similarity analysis to the first 10 listed strains per species in the NCBI metadata file.

### The Bacterial Identification Algorithm MS2Bac

The basic idea of MS2Bac is mapping and counting PSMs from the output of a proteomics search engine to a reference database. In MS2Bac, this mapping can be performed in two iterations. In the first iteration, PSMs are mapped against a species-level reference database, which includes (ideally) all sequenced species of the bacterial domain of life. Species included in the reference database for this study are summarized in Supplemental Table S8. In a second (optional) iteration, the strain-level identity of an isolate can be determined by searching MS2 spectra with a search engine against a strain-level reference database that contains all available strains for the genus identified in the first iteration. Just like in the first iteration, PSMs are mapped to bacteria in the strain-level reference database. This approach was introduced by Kuhring *et al*. (20) and is also implemented in the flash-MS/MS proteotyping approach (51).

The first iteration of the MS2Bac algorithm can be divided into four main steps: (1) reference database construction, (2) 100% FDR search with MSFragger (52) to assign peptide sequences to MS2 spectra, (3) mapping PSMs to bacteria from the reference database, and (4) reporting results. The optional second iteration follows the same four steps.

Creating the reference database for the first iteration must be performed only once and then occasionally when updates are required. We have given great care about the generation of a species-level reference database by selecting from NCBI available type strains per species whenever possible and by considering the following three criteria: (i) NCBI ANI status is “OK” (53), (ii) the species name must be known, (iii) the species taxon contains at least one “representative genome” or a “reference genome” (42). In the case of *Escherichia coli* (species NCBI:taxid:562), two reference genomes were available, and we chose *E. coli* O157:H7 str. Sakai (NCBI:taxid 386,585). In total, 13,855 proteomes from representative strains were downloaded from NCBI (2023-02-17) and *in silico* digested (Supplemental Table S8). Only fully tryptic peptides were allowed. The taxonomic level is defined based on NCBI's taxon identifier (42). For taxon mapping, we applied the Python package ete3 (54). After each iteration, the bacterial species or strain with the most mapped PSMs was identified as the closest taxonomic relative in the database.

In the second step, raw data from liquid-chromatography electrospray ionization tandem mass spectrometry measurements were searched with MSFragger v3.0 (52). Running a single MSFragger search with over 470 million peptide sequences was not feasible on a server with 256 GB RAM, 60 cores, and 120 logical processes due to memory limitations. Instead, running MSFragger with the reference database was only possible when splitting the peptide space into four and performing four individual searches. Default parameters were applied, except that a 100% FDR was used, and quantification was disabled allowing faster run times. MS2Bac combined the four individual searches by selecting the PSM with the highest hyper score per MS2 spectrum. Importantly, false-positive PSMs (typically low quality spectra) randomly matched to peptides across all bacterial species in the reference database and were therefore equally distributed. The advantage of this procedure is that many true positive hits are retained, although a large peptide search space is applied.

In the third step, all PSMs were mapped to taxa. If several species shared a peptide, it was counted several times, but only once per species. The bacterium with the most mapped PSMs was defined as the identified species. However, MS2Bac does not distinguish between isoleucine and leucine, because the mass is identical. Hence, mass-spectrometry is not able to distinguish them.

Lastly, results were reported in a table format and are visualized in a distplot and exported as a single PDF.

In the optional second iteration, all strain-specific proteomes from the identified genus were downloaded from NCBI and combined into a single fasta file. Filtering criteria included the following: (i) NCBI ANI status is “OK,” (ii) the genus must be the same as identified in the first iteration, (iii) the assembly level must be defined as a “Complete Genome.” Once downloaded, MS2Bac will not initiate a new download, except the folder is deleted by the user, for example, if an updated version is required. For the analysis of datasets (v) and (vi) with MS2Bac, we further added strain-specific proteomes for the *Pseudomonas* spp. and *Bacillus* spp. datasets (dataset (v) and (vi)) to the respective database compiled from NCBI. This was necessary because they were not all available on NCBI (Supplemental Table S2). For the analysis with MS2Bac of these two datasets, we used this extended version of the *Pseudomonas* and *Bacillus* database. The genomes of these strains were acquired and assembled in-house. In the case of *Escherichia*/*Shigella*, which are technically the same genus (55), and *Rhizobium/Agrobacterium*, which is suspected to be the same genus (56), we manually concatenated the fasta files. Again, the raw file was searched against the strain-level reference database with MSFragger. The bacterial strain with the most PSMs was defined as the identified subspecies. Of note, we excluded three species of the diversity dataset (i)–(iii) as they were not represented in the reference database: *Bacillus cereus* (no reference or representative genome declared), *Erwinia rhapontici* (no species genome available), and *Kosakonia radicincitans* (no species genome available). In addition to these files, we excluded *Pseudomonas psychrotolerans* and *Pseudomonas mandelii* in the 38 SPD dataset (dataset (ii)) and *Trichococcus collinsii* in the 80 SPD diversity dataset (dataset (iii)), as these samples were picked from the wrong well. In the 38 and 80 SPD diversity datasets, we did not acquire data for *Staphylococcus simulans*. We identified all strains from the 80 SPD diversity dataset at strain level (first + second iteration) with MS2Bac. We manually converted strain names into the DSM or ATCC identifier, for example, with BacDive (57) or using a literature research (Supplemental Table S2) and checked the identification rank. If the first rank had no strain designation, we interpreted the second as rank one. Dairy product isolates were identified with a first and second iteration, while clinical isolates were identified with a first iteration only.

Ten isolates of the clinical dataset were searched against a fungal strain-level reference database. Database construction and MS2Bac analysis was performed identical to the procedures described for strains and isolates of bacterial origin, but for fungal origin. A list of these 584 strains is provided in Supplemental Table S9.

MS2Bac's source code is freely available as described in the Data availability paragraph. MS2Bac utilizes, among others (see MS2Bac_conda_environment.yml), the following packages: Pyteomics (58), pandas (59), numpy (60), seaborn (37), and matplotlib (36).

## Results

### A Proteomic Resource Across the Bacterial Domain of Life

**Fig. 1:**
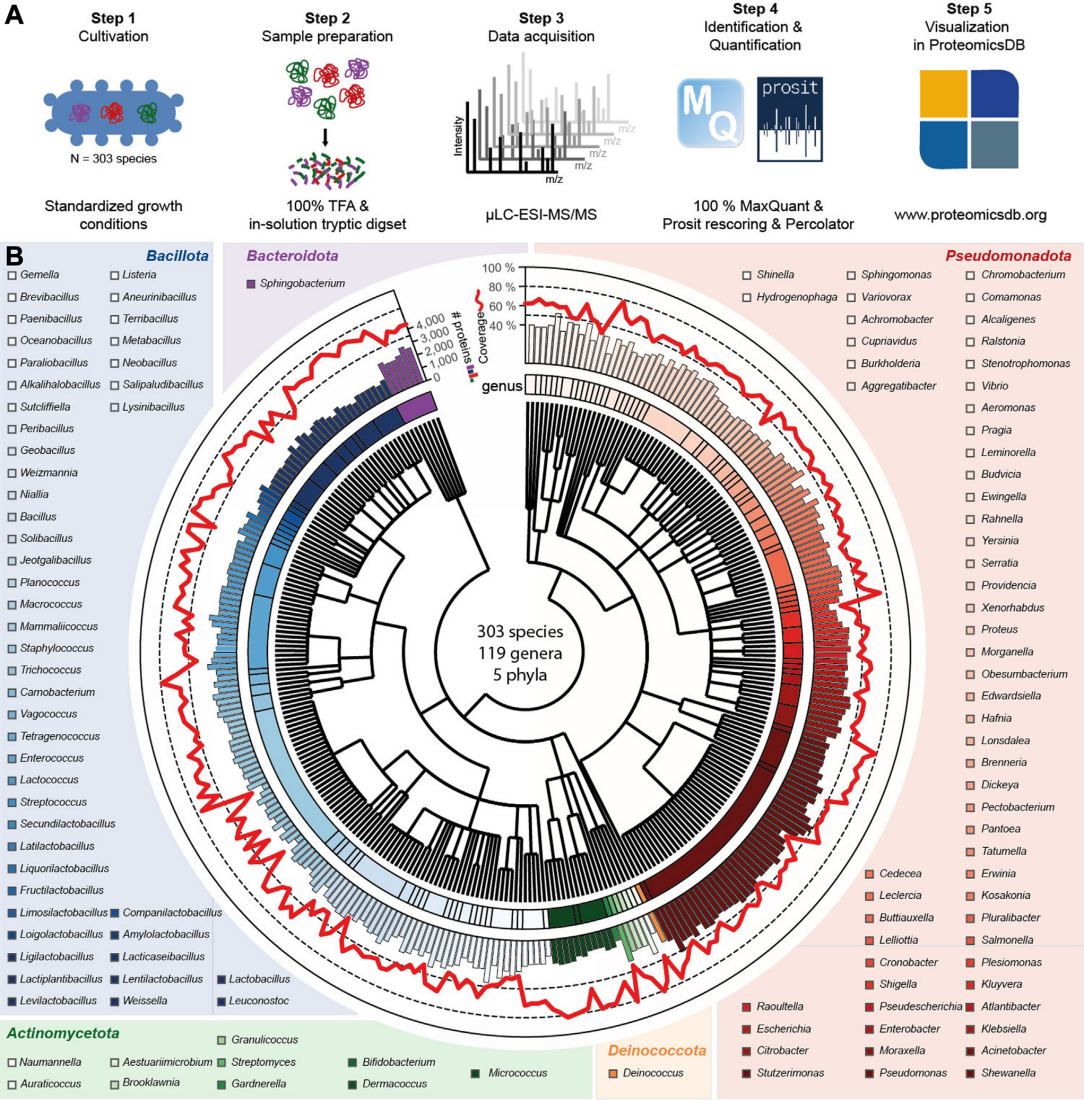
**Proteome profiles across the bacterial domain of life**. *A*, illustration of the applied workflow: Bacteria were grown under standardized conditions (Supplemental Table S2). All bacteria were lysed with 100% TFA and subjected to in-solution tryptic digestion. Mass spectrometric measurements and data analysis were performed using state-of-the-art instrumentation (microflow liquid chromatography system coupled to an Orbitrap Exploris 480 mass spectrometer) and software (MaxQuant combined with Prosit rescoring). All proteomes are freely accessible and can be interactively browsed on ProteomicsDB (https://www.proteomicsdb.org/). *B*, overview of the proteomes across the bacterial domain of life. Bacteria were taxonomically classified according to the National Center for Biotechnology Information (NCBI). The standard taxonomy tree represents the bacterial relationship. Colors represent genera. Protein identifications at 1% FDR per organism are depicted as bars. The proteome coverage, relative to all ORF-encoded genes per species, is shown as a *red line*. If more than one strain per species was available, the strain with the highest protein numbers is displayed.

We present a comprehensive proteomic expression landscape for 318 bacterial strains annotated to 303 species, 119 genera, and five phyla (Supplemental Fig. S1, *A* and *B*) analyzed with a unified bacterial workflow (Fig. 1*A*; (7)). The two most diverse phyla represented in the dataset are Bacillota and Pseudomonadota, which account for >90% of all species (Supplemental Fig. S1*B*).

The proteomic resource presented here corroborates the expression of 636,558 unique proteins (Fig. 1*B* and Supplemental Fig. S1, *C* and *D*). Around 94% of the detected proteins were backed up by at least two peptides, and any given protein had, on average, 12 representative peptides (Supplemental Fig. S1*E*). Since bacterial proteomes vastly differ in size, the absolute number of detected proteins ranged between 476 (*Staphylococcus cohnii*) to 3736 (*Variovorax paradoxus*) (Fig. 1*B*). We could stably detect over 50% of the theoretical genome-coded proteome for most species (Fig. 1*B* and Supplemental Fig. S1*F*). The presented resource meets or exceeds the proteomic depth of other proteome profiling efforts (Supplemental Fig. S1*G*; (6, 7, 61). A popular database, Uniprot/SwissProt (https://www.uniprot.org/, accessed 14 October 2024) (50), reports levels of evidence for each ORF in a genome. The highest level is “experimental evidence at protein level,” which has not yet been reported for most proteins detected in the experimental proteomes published in this study (630,000 proteins). Hence, this study increases the current SwissProt knowledge by 26-fold and SwissProt/TrEMBL by 15-fold. According to NCBI (https://www.ncbi.nlm.nih.gov/NCBI) (3), 11% (n = 125,758) of all proteins encoded in the genomes of the 318 strains are annotated as “hypothetical” (no biological function *via* homology transfer). In this study, protein-level evidence could be found for 31% (38,766) of those hypothetical proteins (Supplemental Fig. S1*H*), prioritizing them for further experimental studies.

The experimental proteomes have been incorporated into ProteomicsDB, a multiorganism resource for life science research (https://www.proteomicsdb.org/; (8)). ProteomicsDB enables a straightforward visual exploration of the data to find experimental proof for any peptide or protein of interest and serves as a repository for targeted proteomics assay development. As this project is the first to include single-cell organisms in ProteomicsDB, alternative visualizations and advanced selection modules have been introduced to enhance the exploration of the comprehensive list of organisms (Supplemental Fig. S2, *A*–*C*).

### Quantitative Exploration of Proteomes from 303 Bacterial Species

The quantitative nature of our MS-based proteomics dataset enabled us to report evidence for protein expression and explore protein abundance levels within and across bacterial species. Thus, we z-scored iBAQ (33), leading to consistent protein expression distributions between species (Supplemental Fig. S3, *A* and *B*). The quantitative data is visualized within ProteomicsDB (Supplemental Fig. S2*D*). The relative iBAQs were comparable to PaxDB (62), a database for absolute protein abundances (Supplemental Fig. S3*C*). The determined protein abundance ranges from the 303 bacterial species covered four to six orders of magnitude with no bias towards proteome size (Supplemental Fig. S3, *D*–*E*). On average, only 3% of the highest expressed proteins contributed to 50% of the total protein intensity (Supplemental Fig. S3*F*). Essential cellular protein functions like “structural molecule activity” or “structural constituent of ribosome” were enriched among the top 10 proteins per species (Supplemental Table S7). Surprisingly, of the top 10 most abundant proteins, 5% (n = 163/3148) were classified as “hypothetical proteins” (Supplemental Fig. S3*E* and Supplemental Table S10). As high protein abundance often signifies functional relevance, these highly abundant hypothetical proteins present particularly intriguing targets for further experimental studies.

**Fig. 2:**
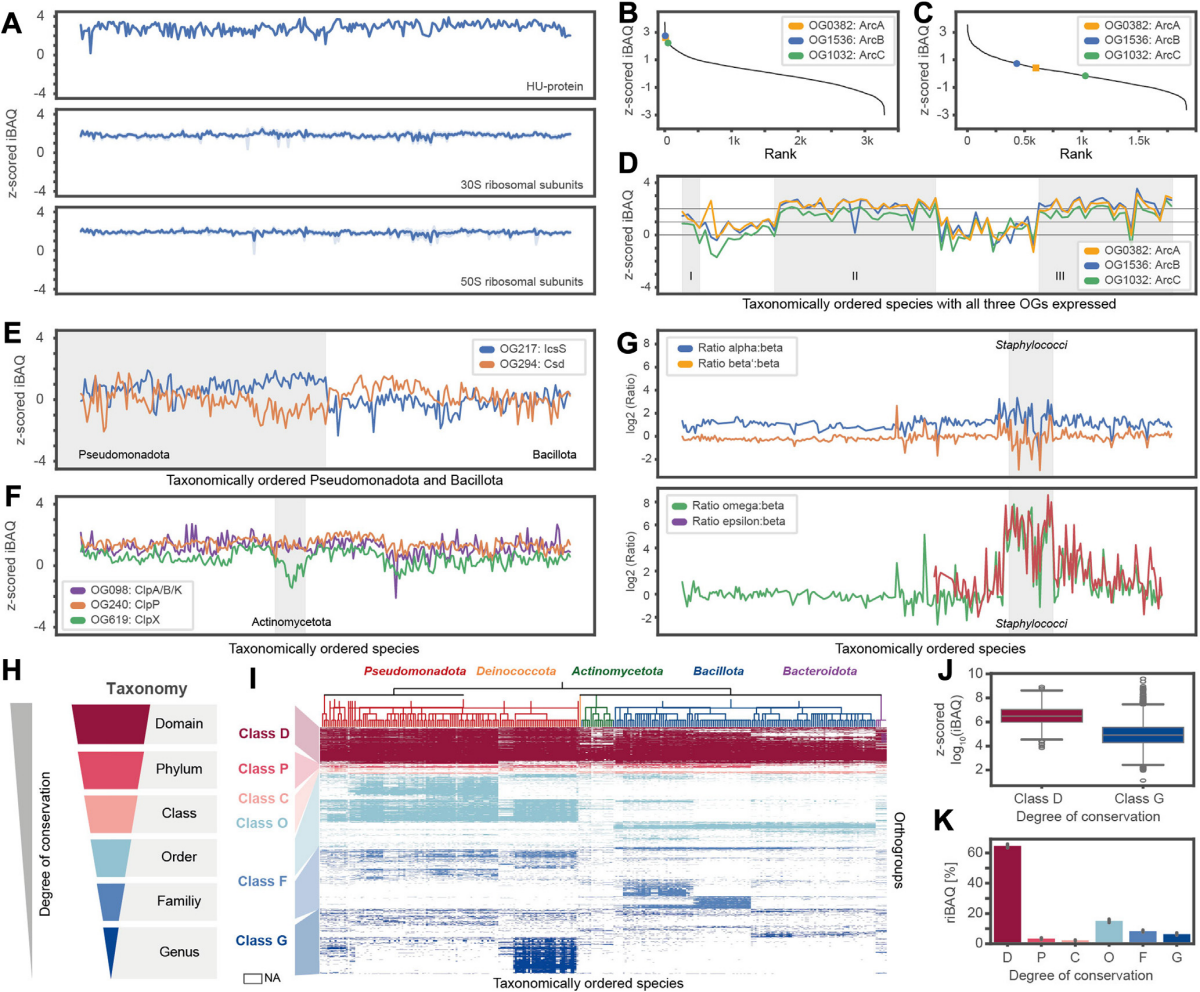
**Orthogrouping enables cross-species protein abundance comparisons**. *A*, proteins from orthogroups (OGs) that were conserved and consistently highly expressed across all characterized bacterial species, including DNA-binding protein HU (OG129), proteins of the 50S and the 30S ribosomal subunits. Their z-scored protein intensity values (iBAQ) are depicted in taxonomical order. *B*, ranked protein expression plots with z-scored iBAQ values of ArcA (*blue*), ArcB (*yellow*), and ArcC (*green*) from *Pseudomonas aeruginosa*. Only the highest intense paralog within a species' OG is considered. *C*, same as (*B*), but for *Bacillus cereus*. *D*, same as (*a*), but for the OGs of ArcA (OG1536), ArcB (OG382), and ArcC (OG1032) across species where they were all successfully quantified. Only the highest intense paralog within a species' OG is considered. Taxon cluster I: Chromobacteriaceae, Rhizobiaceae, Vibrionaceae; cluster II: Pseudomonadaceae; cluster III: Enterococcaceae, Streptococcaceae, Lactobacillaceae. *E*, same as (*A*), but for OGs of cysteine desulfurases. *F*, same as (*A*), but for the ATP-dependent proteases ClpP (OG240), ClpX (OG619), and ClpA/B/K (OG098). *G*, same as (*A*), but for OGs representing RNA-polymerase subunits. *H*, schematic representation of the taxonomic hierarchy. The applied color code is used for subfigures (*H*–*K*). *I*, OGs were classified according to their taxonomic conservation into six classes: domain (D), phylum (P), class (C), order (O), family (F), and genus (G). Only OGs that contain proteins from more than 20 species are shown. Species whose reference proteome does not encode a protein of a given OG are *left* blank. OGs were colored according to membership to one of the six classes expressing taxonomic conservation. *J*, boxplots comparing z-scored iBAQ intensities from quantified proteins belonging to OGs classified as class D *versus* OGs classified as class G. *K*, summed relative iBAQs (riBAQ) of quantified proteins per species split across the six classes of taxonomic conservation. Error bars represent the 95% quantile (default in seaborn) between species.

Next, we grouped proteins into OGs to enable protein abundance comparisons across species. Around 97% of all genome-coded proteins (n = 1,131,620) were assigned to 34,979 OGs (Supplemental Table S6). Intriguingly, only 99 OGs were present in all analyzed strains without exception (Supplemental Fig. S4*A*). Housekeeping proteins such as the DNA-binding protein HU (OG129), the 33 different 50S, or the 21 different 30S ribosomal subunits were highly and stably expressed across all 318 strains (Fig. 2*A*). We implemented a cross-species visualization module in ProteomicsDB which integrates the intricate outputs of ortholog mapping and quantitative proteomics. It provides users with an interactive visualization combining both elements in one comprehensive view (Supplemental Fig. S2*E*).

Not all OGs were consistently expressed across species, but they showed interesting taxon-specific regulations. For example, for the *arcDABC* operon, we could confirm the anticipated higher expression of ArcA (arginine deiminase) and ArcB (ornithine carbamoyltransferase) compared to ArcC (carbamate kinase) in *P. aeruginosa* (Fig. 2*B*) (63). The arginine-ornithine antiporter (ArcD) was inconsistently detected by MS-based proteomics, most likely because this is a highly hydrophobic membrane-bound protein with unfavorable properties for mass spectrometry (64). A similar quantitative protein expression pattern could be observed for the *arcDABC* operon in *B. cereus*, however, at a generally lower abundance level relative to the rest of the proteome (Fig. 2*C*). Taking into account all species investigated in this study, protein expression levels of ArcA (OG1536), ArcB (OG382), and ArcC (OG1032) were regulated in a taxon-specific way (Fig. 2*D*). In Pseudomonadacae, Enterococcaceae, Streptococcaceae, and Lactobacillaceae, all three proteins were around two sigma higher expressed than the mean. In Chromobacteriaceae, Rhizobiaceae, and Vibrionaceae, they were expressed at around 1 to 1.5 sigma higher than the median. In contrast, in all other taxa, they were expressed at an average proteome level (Fig. 2*D*). This suggests that Pseudomonadacae, Enterococcaceae, Streptococcaceae, and Lactobacillaceae depend more on arginine catabolism, thus allocating a significant proportion of their energy to producing these enzymes.

Another example of a taxon-specific expression pattern is the protein cysteine desulfurase (LcsS, OG217), an essential protein in the Fe-S cluster assembly which is highly conserved in bacteria and eukarya. This cysteine desulfurase was around one sigma higher expressed in Pseudomonadota than in Bacillota. Intriguingly, an alternative cysteine desulfurase (Csd, OG294) showed an opposing expression pattern (Fig. 2*E*), indicating a phylum-specific regulation of the Fe-S cluster assembly in bacteria. The ATP-dependent protease ATP-binding subunit ClpX (OG619) was significantly lower expressed in Actinomycetota, with *Bifidobacterium* spp. having the lowest expressing levels (Fig. 2*F*). Another example of a taxon-specific expression pattern is the DNA-directed RNA polymerase, which consists of five subunits (core: ααββ'; accessory: ωσ; ε in gram-positive bacteria (65)). We observed that α, β, and β′ subunits were stably expressed at the expected 2:1:1 ratio (www.ebi.ac.uk/complexportal/; (66)) throughout all investigated species (Fig. 2*G*). In contrast, subunits ω and ε were 2- to 6-fold higher expressed in all 32 species of Staphylococcaceae.

Many of the most highly expressed proteins in the dataset showed a high degree of conservation between species. To test if a correlation between protein expression and conservation exists, we classified all OGs according to their taxonomic conservation on the level of domain (class D), phyla (class P), classes (class C), orders (class O), families (class F), or genera (class G) (Fig. 2*H* and Supplemental Fig. S4, *B* and *C*). We found that ∼1000 OGs, consisting of ∼307,000 proteins, were conserved across the bacterial domain (class D, they do not have to be present in each species, but across all phyla), while ∼10,000 OGs were conserved on a genus level (class G) (Supplemental Fig. S4, *D* and *E*). Based on this classification scheme, we found that conserved proteins (class D) were 1.6 sigma more abundant than genus-specific proteins (class G) (Fig. 2*J* and Supplemental Fig. S4*F*). Interestingly, class D proteins contributed over 60% to a bacterium's overall protein intensity (Fig. 2*k*).

### Bacterial Peptidomes are Highly Distinct

Tryptic peptide sequences can be shared within and across bacterial species, affecting confident protein identification and quantification (14, 18). We calculated tryptic peptide overlaps between the 318 characterized strains to investigate the extent of peptide sharing. Surprisingly, almost 40% of all detected tryptic peptides (n = 3,893,376) uniquely mapped to only one of the 318 strains (Supplemental Fig. S5). Extending this analysis to all theoretical tryptic peptides derived from the genomes of the 318 strains and eventually to one representative strain per species in NCBI (n = 13,855) yielded similar findings: On average, over 50% of the tryptic peptidome could be uniquely assigned to one specific bacterial species (Supplemental Fig. S5). Nonetheless, closely related species within the same genus shared more of their tryptic peptidomes than phylogenetically more distinct species. Specifically, pair-wise comparisons of species within the same genus had median overlaps of 7.6%, 3.4%, and 4.9% for Pseudomonadota, Actinomycetota, and Bacillota, respectively (Supplemental Fig. S6, *A*–*C*). In contrast, species from different genera of the same phylum had median overlaps of only 0.2%, 0.2%, and 0.4%, respectively. This theoretical analysis demonstrated a surprisingly high diversity in bacterial peptidomes. Furthermore, proteomic similarities reflect the taxonomic relationship: Closely related species had higher Jaccard coefficients than distinct species (Supplemental Fig. S6, *D*–*F*).

Next, we examined the similarity of tryptic peptidomes at strain level. As expected, the peptide overlap was higher than at the species level, with median overlaps of 73.3% for *Pseudomonas* spp. and 70.5% for *Bacillus* spp. (Supplemental Fig. S6, *G* and *H*). Despite this high similarity, every 25th tryptic peptide is still uniquely mapped to one specific bacterial strain, enabling the discrimination of even very closely related bacteria at both the species and strain levels.

### MS2Bac: A Simple Bacterial Identification Algorithm

**Fig. 3:**
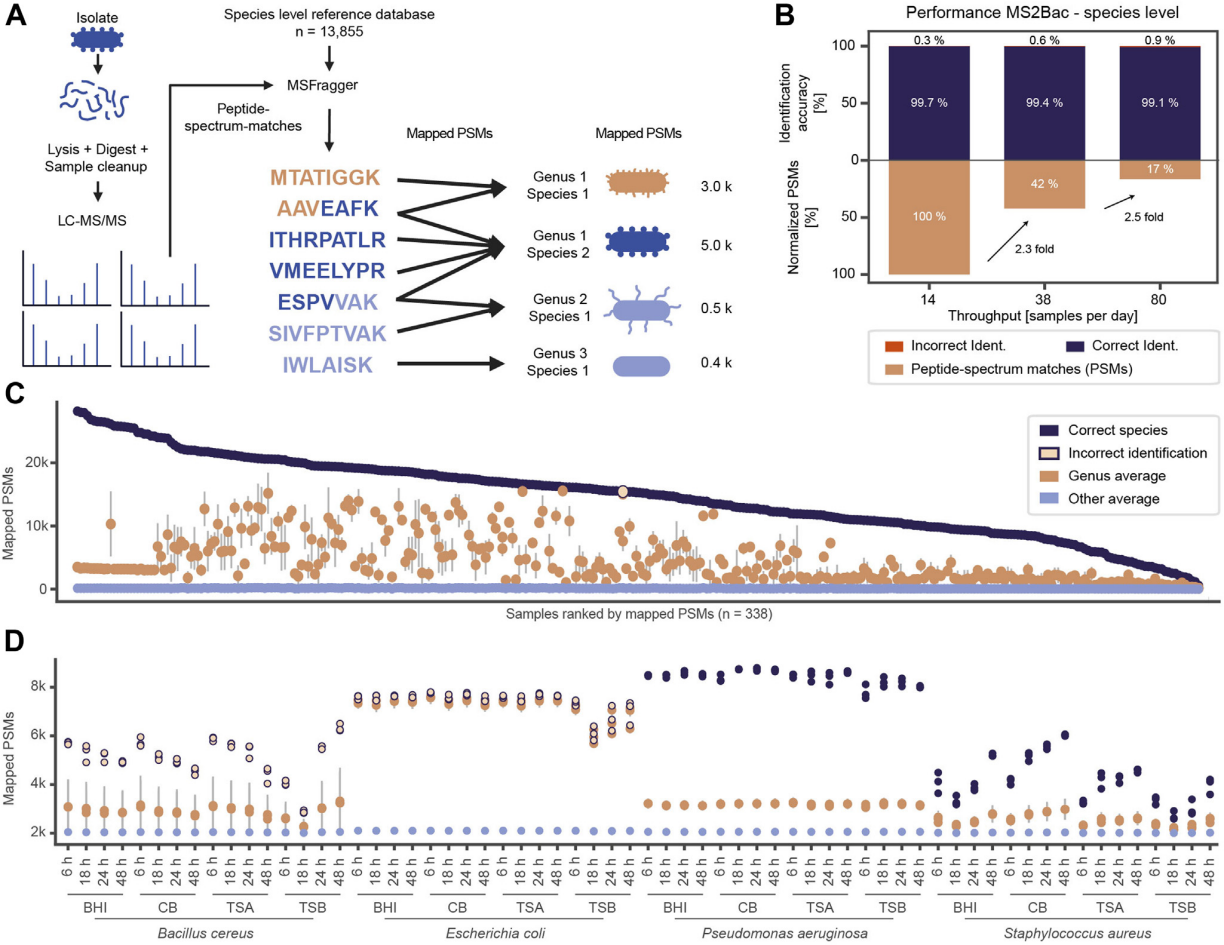
**MS2Bac—a simple bacterial identification algorithm based on mass spectrometric proteomics data**. *A*, schematic depiction of the MS2Bac workflow: Pure cultures are lysed, proteins are digested with trypsin, and peptides are measured using MS-based proteomics. Peptide-spectrum matches (PSMs) are determined with MSFragger against a species-level reference database. The reference database contains 13,855 bacterial species currently deposited in NCBI. The sample of interest is identified as the species with the most mapped PSMs. *B*, MS2Bac shows a correct identification accuracies of 99% (*blue*) on the species-level when applying the 14 samples per day (SPD) method. *Red* represents incorrect identification. *Beige* bars represent relative numbers of PSMs from correctly identified samples. *C*, the mapped PSM count from the 14 SPD diversity dataset follows the phylogenetic relationship of bacteria: The species with the most mapped PSMs (correctly identified in *dark blue*, incorrectly identified in *beige-blue*) is followed by other species of the same genus (average genus-level PSM is shown in *beige* and SD as a *gray* bar). Species of different genera accumulate at low PSMs (average PSM in *light blue*). *D*, reproducibility tested on four different species grown in triplicates under various conditions: Columbia blood agar (CB), brain-heart infusion agar (BHI), tryptic soy agar (TSA), and tryptic soy broth (TSB) at four different time points (6 h, 18 h, 24 h, and 48 h). Same color scheme as (*C*).

Inspired by the distinct tryptic peptidomes of bacteria, we developed a simple bacterial identification algorithm called MS2Bac (Fig. 3*A*). We first created a comprehensive species-level reference protein database for species-level identifications by selecting one representative strain for each bacterial species deposited in NCBI (status as of 03 March 2023). This resulted in a reference database comprising 13,855 species, 2897 genera, and 40 phyla (Supplemental Fig. S7, *A* and *B*) and consisted of 484,142,246 de-duplicated tryptic peptides. Next, we used this reference database together with a state-of-the-art database search tool (MSFragger, (52)) to determine PSMs. We did not apply any FDR control because MS2Bac neither claims nor reports the existence of individual peptides but exclusively serves as a taxon identification tool (Supplemental Fig. S7, *C* and *D*). Without an FDR control, the approach is scalable, because true peptide identifications are not sacrificed for a stringent FDR cutoff with large databases. This is justified because PSMs accumulate for the correct species, resulting in high PSM counts, while incorrect matches are evenly distributed among all other species, keeping their PSM counts low (Supplemental Fig. S7, *C* and *D*). Since taxonomically unrelated species share only a small fraction of peptides, they can be considered as “decoy” species, similar to the target-decoy approach used in LC-MS/MS-based proteomics for identifying fragment ion spectra (67). Finally, MS2Bac counted the number of PSMs for each species in the reference database and identified the species with the most mapped PSMs. More details about the algorithm can be found in the Experimental Procedures section.

To validate the performance of MS2Bac, we applied it to our dataset of 303 species. In all but one case, MS2Bac consistently mapped the highest number of PSMs to the correct species, resulting in an identification accuracy of >99% (Fig. 3*B* and Supplemental Table S2). Closely related species from the same genus provided the second-highest number of PSMs, while low PSM counts (111 PSMs on average) were assigned to other genera (Fig. 3*C* and Supplemental Fig. S7*E*). Only one sample was misidentified: *Shigella boydii* as S. *sonnei* (Supplemental Fig. S7*E*, middle panel). In this specific case, the sample strain *S. boydii* DSM7532 had a more similar *in silico* tryptic peptidome to the representative strain *S. sonnei* SE6-1 (60% peptide identity) than to the representative strain *S. boydii* ESBL-W3-2 (58% peptide identity) entailed in the reference database. Consequently, MS2Bac assigned most PSMs to *S. sonnei*. In general, species of the genus *Shigella* spp. and *E. coli* show very high taxonomic relatedness (overlaps >50%), supporting prior proposals in the literature for their reclassification into a single species (55, 68).

### MS2Bac is Robust Against Data Acquisition and Variances in Bacterial Growth

High sample throughput and robustness against variations in growth conditions and sample preparations are crucial prerequisites for routine diagnostic applications. To test MS2Bac's performance, we generated a dataset with increased sample throughput from initially 14 SPD to 38 SPD and eventually 80 SPD. Additionally, we shortened the tryptic digestion time from overnight to 2 hours, enabling the sample preparation workflow to be completed within a single working day. While these adaptations substantially reduced recorded spectra and PSM counts per species, the identification accuracy still exceeded 99% (Fig. 3*B*, Supplemental Fig. S7, *F* and *G*, Supplemental Table S2). In addition to the misidentification of *S. boydii* described above, MS2Bac incorrectly identified *Serratia marcescens* as *S. nematodiphila* in the 80 SPD dataset, again due to a high tryptic peptide overlap between the tested strain and the two reference species (tryptic peptide overlaps in both cases 62%).

Next, we tested MS2Bac's robustness to varying growth conditions, a known vulnerability with other identification methods, such as MALDI-TOF (69). For this experiment, we selected four representative species: *B. cereus*, *P. aeruginosa*, *Staphylococcus aureus*, and *E. coli*, and grew them for 6, 18, 24, and 48 h on three different agars and in liquid culture. The number of PSMs and the identification results obtained by MS2Bac were stable over time and across all tested growth media (Fig. 3*D*). *B. cereus* was consistently (mis-)identified as *B. thuringiensis* because *B. cereus* was a species not included in the reference database due to a missing reference/representative proteome. *E. coli* was consistently (mis-)identified as *S. sonnei*. As discussed above, these two species are taxonomically close relatives which can be differentiated through a second analysis step as described below. Although PSM numbers were constant, we observed significant quantitative differences in the protein expression profiles from different growth conditions (Supplemental Fig. S8) and culturing times (Supplemental Fig. S9). Nevertheless, MS2Bac remained unaffected by these variations and reported a consistent number of mapped PSMs per species, thereby enabling accurate identification regardless of the applied growth conditions (Fig. 3*D*).

### MS2Bac Allows Bacterial Identification at Strain-Level

**Fig. 4:**
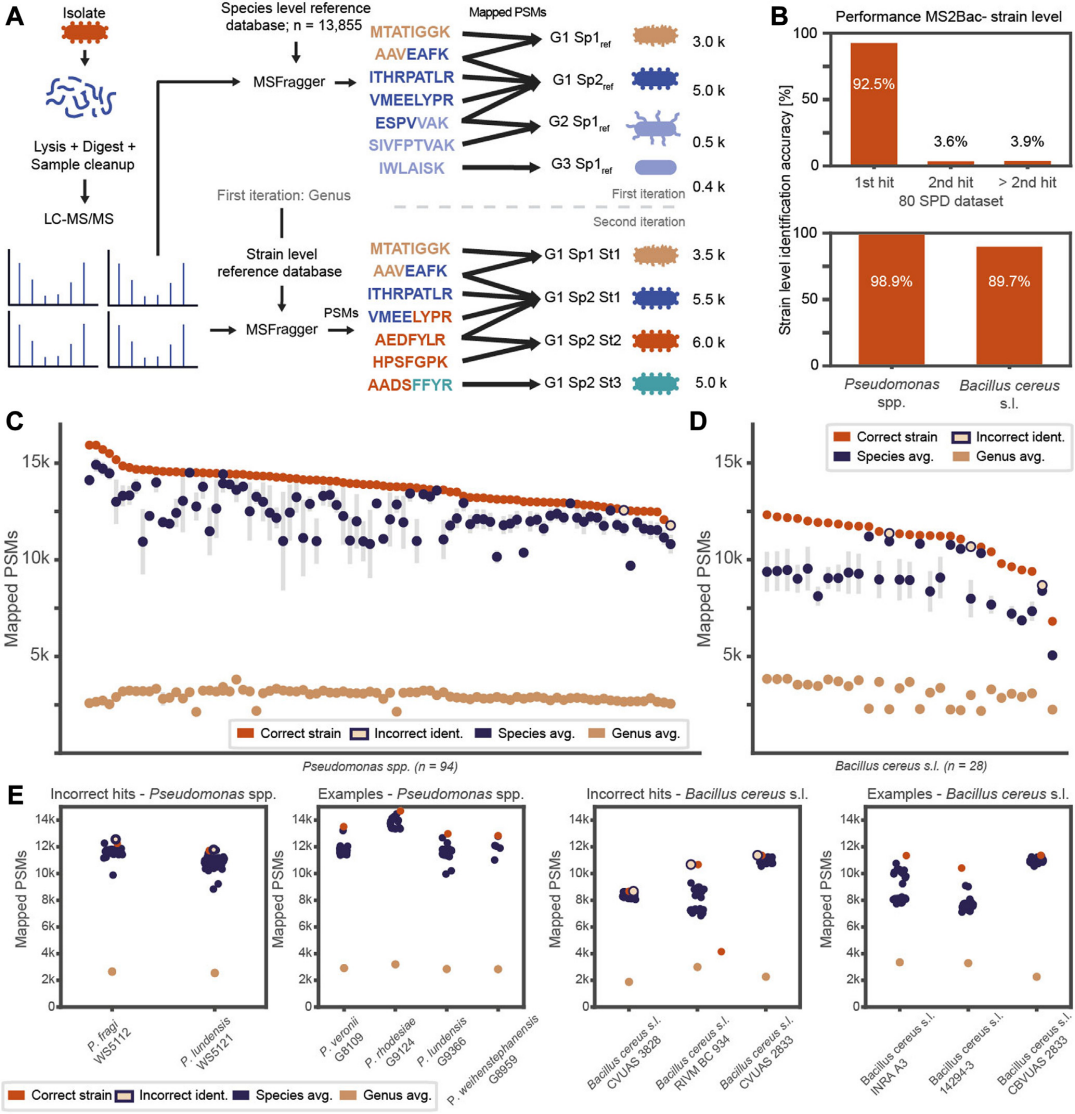
**MS2Bac facilitates bacterial identification at strain level**. *A*, schematic depiction of the workflow for strain-level bacterial identification. The first iteration follows the workflow presented in Fig. 3. A second iteration is needed for strain-level identification. The identified genus from the first iteration is used to download all strains of this genus from NCBI. This strain-level reference database is then used for the MSFragger search in the second iteration. The sample of interest is identified as the strain with the most mapped PSMs. *B*, performance evaluation of MS2Bac at strain-level. The datasets used to benchmark MS2Bac's capabilities for strain-level identification entailed 335 strains from the 80 SPD dataset, 94 strains from the genus *Pseudomonas*, and 28 strains from *Bacillus cereus s.l.* In the 80 SPD dataset, the identification rank is depicted. For the other two datasets, only rank one hits are depicted. *C*, the number of PSMs in the *Pseudomonas* dataset follows the phylogenetic relationship of bacteria. The strain with the most mapped PSMs is in 93 out of 94 samples correctly identified (*red*), followed by other strains of the same species (average species-level PSMs in *dark blue* and SD as *gray* bar). Strains from different species accumulate at low PSMs (average PSMs in *orange*). The incorrect strain identification is shown in *beige* with a *blue* frame. *D*, same as (*C*), but for *Bacillus cereus* s.l. *E*, examples of correctly and incorrectly identified strains from the *Pseudomonas* and *Bacillus cereus* datasets. Correctly identified strains are indicated in *red*; incorrectly identified strains are in *beige* with a *blue* frame. Every *dark blue* dot represents a strain from the same species, while the single *orange* dot depicts the averaged PSM count from strains of different species.

In the theoretical analysis of overlapping tryptic peptidomes, we found that they are surprisingly distinct at the (sub-)species levels (Supplemental Fig. S6, *G* and *H*). We included a second iteration analysis step into MS2Bac, similar to the approach introduced by Kuhring *et al*. (20) (Fig. 4*A*). Based on the result from the first iteration, the MS2Bac algorithm compiles all available NCBI proteomes for the identified genus into a strain-specific reference database. This database is then used to search the data and determine the strain with the most mapped PSMs. The size of the strain-specific reference database for the second iteration varied for each genus, depending on the number of species and strains deposited in NCBI (Supplemental Fig. S10). We analyzed the 80 SPD dataset and additionally acquired data for 94 individual strains of the genus *Pseudomonas* spp. and 28 strains of the *B. cereus* s.l. group. Species from these two genera are challenging to identify with other methods due to their phylogenetic relatedness (9). MS2Bac achieved a correct strain-level identification accuracy of 92.5% (n_80 SPD dataset_ = 311/336), 98% (n_*Pseudomonas*_ = 87/89), and 89% (n_*Bacillus*_ = 25/28), respectively (Fig. 4, *B*–*E*). In the 80 SPD dataset, another 12/336 were ranked second (Fig. 4*B*). Interestingly, the second iteration corrected the other two misidentifications from the first iteration, *S. marcescens* and *S. boydii*. This can be explained by the nature of the species-level reference database from the first iteration, which contains only one representative strain per species. However, if this strain does not optimally represent the analyzed strain, misidentifications can be corrected in the second iteration if the reference database contains information on the given strain. Another example is *B. cereus* that was not present in the first iteration reference database and was therefore consistently misidentified as *B. thuringiensis* (Fig. 3*D*). It is important to note that the first iteration reference can be updated with a nonreference strain of *B. cereus*. In the second iteration, this identification result was corrected, because all deposited strains, including *B. cereus* proteomes, were included. In conclusion, a second iteration of MS2Bac analysis provides more accurate identification at both the species and strain level.

### Merits of MS2Bac for Bacterial Identifications from Food-Derived Samples

**Fig. 5:**
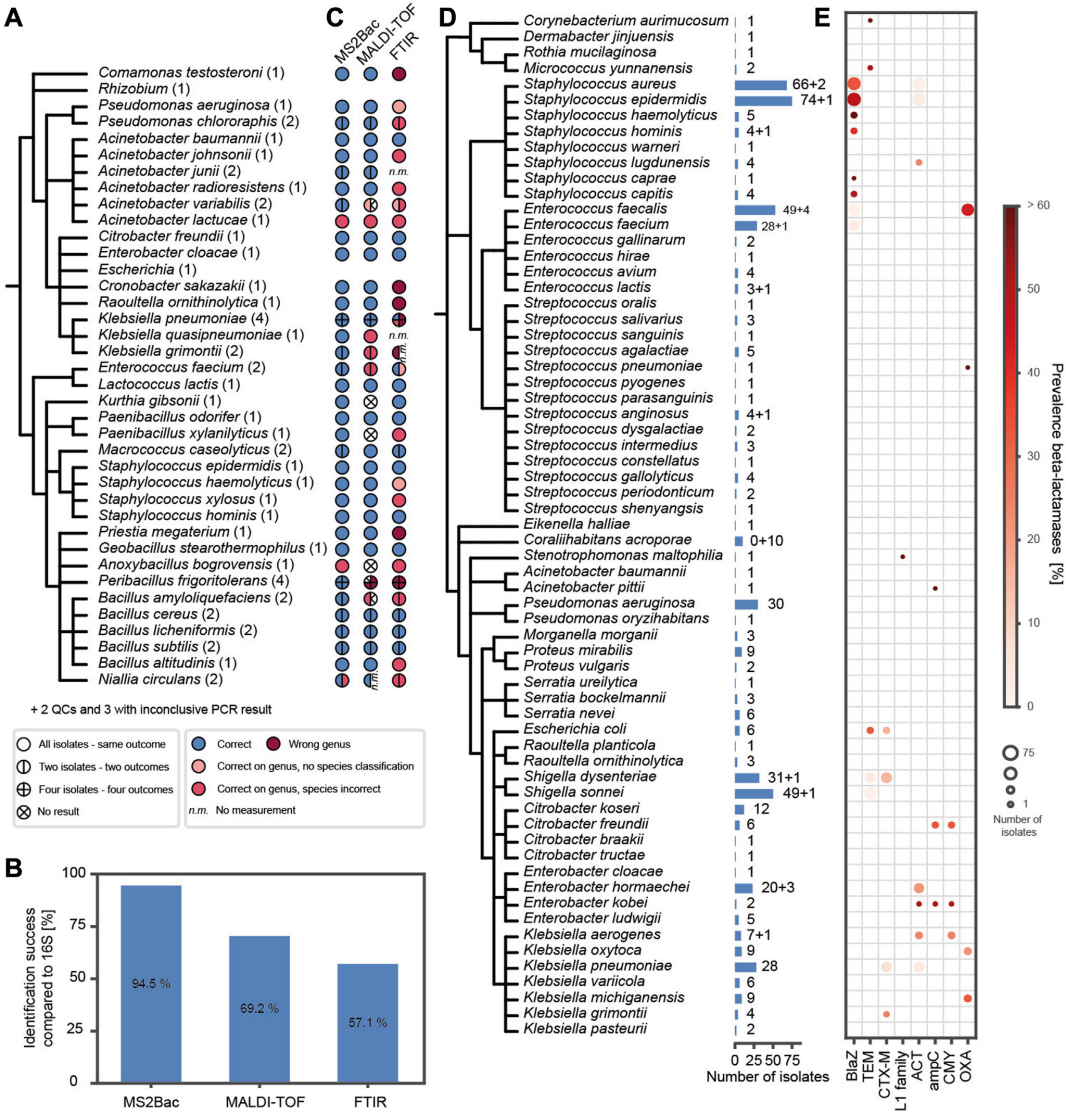
**MS2Bac for bacterial identifications from food-derived and clinical samples**. *A*, common taxonomy tree (NCBI) constructed based on the results from 16S rRNA gene sequencing, delineating 39 bacterial species identified from 60 isolates of dairy products. *B*, global identification accuracy of MS2Bac with a first and second iteration, MALDI-TOF MS, and FTIR compared to 16S rRNA gene sequencing. *C*, MS2Bac, MALDI-TOF MS, and FTIR identification success compared to 16S rRNA gene sequencing. Species-level accordance is denoted in *blue*. Correct genus-level without species identification is indicated in *orange*. Genus-level with incorrect species identification is shown in *light red*. Incorrect genus-level identification is marked in *dark red*. Some MALDI-TOF spectra did not yield a high-quality spectrum match (‘No result’). *D*, common taxonomy tree (NCBI) constructed for 64 bacterial species identified from 570 clinical isolates received from a clinical diagnostics laboratory for medical microbiology, utilizing MS2Bac and a first iteration. A horizontal bar chart represents the number of identified isolates per species. The second number behind the bar indicates isolates that did not meet quality control standards (*e.g.*, insufficient organic material, losses during sample preparation, less than 500 mapped PSMs). *E*, expression pattern of selected beta-lactamase antibiotic resistance marker proteins. The size of each circle corresponds to the total number of isolates detected for a given species. Color coding indicates the prevalence of the respective beta-lactamase, reflecting the percentage of bacterial isolates expressing the given beta-lactamase.

Next, we explored the merits of MS2Bac as a diagnostic tool for food analysis. We applied MS2Bac to 58 pure bacterial cultures isolated from dairy products from a routine diagnostic laboratory and two QC samples (*P. aeruginosa*). They were compared to 16S rRNA gene sequencing (=reference), FTIR spectroscopy, and MALDI-TOF mass spectrometry. For 55/58 isolates, a 16S rRNA gene sequencing result was retrieved. These isolates were classified into 39 species from 23 genera (Fig. 5*A*). MS2Bac showed the highest accordance with 16S rRNA gene sequencing, correctly identifying 52/55 isolates at the species level (94%; Fig. 5, *B* and *C*). However, *Acinetobacter lactuacae*, *Anoxybacillus bogrovensis*, and one *Nialla circulans* isolate were misidentified as *Acinetobacter pittii*, *Anoxybacillus karvacharensis*, and *Niallia alba* at the species-level. Correct identification for *A. bogrovensis* was impossible with MS2Bac due to the absence of this species in the NCBI database. MALDI-TOF mass spectrometry misidentified 11 samples, while another seven measurements did not yield high-quality hits. Consequently, the correct identification rate dropped to 69% (Fig. 5, *B* and *C*). A correct species-level identification with MALDI-TOF was impossible for 11 isolates from six species due to an incomplete experimental spectrum database. FTIR spectroscopy showed the lowest accuracy, with only 57% correct identifications at the species-level (Fig. 5, *B* and *C*). Interestingly, in the case of *A. lactucae* identified by 16S rRNA sequencing, the other three methods consistently identified it as *A. pittii*. This discrepancy raises severe doubts about the accuracy of the 16S rRNA sequencing result for this species.

### MS2Bac for Clinical Diagnostics and Detection of Antibiotic Resistance

We received 570 pure isolates from a clinical diagnostics laboratory for medical microbiology. MS2Bac successfully identified 64 bacterial species from 20 genera and three phyla (Fig. 5*D*). Notably, prevalent species typically encountered in clinical settings included *Shigella* spp.*/E. coli* (15.4%), *Staphylococcus epidermidis* (13.2%), *S. aureus* (11.9%), and *Enterococcus faecalis* (9.3%) (Fig. 5*D*). Twenty-seven samples from this dataset required exclusion from MS2Bac analysis due to various reasons: insufficient organic material on the agar plate (n = 8), losses during sample preparation (n = 7), or yielding very few PSM assignments (n = 12; PSMs <500). Among the 12 isolates with notably low mapped PSMs, 10 were consistently classified as *Coraliihabitans acroporae*. Suspecting fungal origin, we conducted a second iteration analysis against a reference database, including all available fungal proteomes deposited in NCBI (n = 584, as of 08 May 2024). This revealed an increase in mapped PSMs from <80 to >3498, confirming our hypothesis. We identified fungal species commonly involved in human infections of immunocompromised patients, like *Nakaseomyces glabratus* (2), *Candida albicans* (4), *Candida parapsilosis* (3), and *Pichia kudriavzevii* (1) (Supplemental Table S2).

In addition to MS2Bac 's capability for bacterial identification, the acquired mass-spectrometric data can also be used to identify and quantify specific proteins of interest. For instance, detecting the expression of proteins responsible for antibiotic resistance aids in identifying (multi-)resistant strains. As proof of principle, we re-analyzed the data from all isolates by searching exclusively against the previously identified reference proteome, which was appended with sequences of antibiotic resistance proteins from the Comprehensive Antibiotic Resistance Database (n = 4774; https://card.mcmaster.ca/ (38)). Stringent protein identification control was applied. This approach successfully identified 146 clinically relevant antibiotic resistance markers, including drug-efflux pumps, chloramphenicol acetyltransferases, and beta-lactamases. Remarkably, beta-lactamases were detected in 26% of all clinical isolates (n = 146/560, Supplemental Fig. S11). In particular, a high prevalence of beta-lactamase Z expression in isolates from the genus *Staphylococcus*, which hydrolyzes the beta-lactam ring of penicillins, was apparent (Fig. 5*E*). Additionally, extended-spectrum beta-lactamases (ESBLs), such as TEM, CTX-M, or OXA, were detected in isolates from various genera (*Shigella, Klebsiella, Escherichia, Enterococcus*; Fig. 5*E*). The rise of antimicrobial resistance, particularly from ESBL-producing Enterobacteriaceae, poses a significant global health challenge for which rapid and specific examination methods are urgently needed (70).

## Discussion

This quantitative proteome resource covering 303 bacterial species confirmed the expression of over 625,000 proteins and stably detected more than 50% of the theoretical genome-encoded proteome per species. Numerous “hypothetical” proteins were among the most highly expressed, suggesting an unexplored realm of protein functionality in bacteria. This resource can prioritize abundant, yet functionally unknown, proteins for further studies. To facilitate visual exploration for the community, we integrated the data into ProteomicsDB (https://www.proteomicsdb.org/). Here, we introduced a new OG-visualization module, enabling protein abundances comparisons across species. Our quantitative OG-based analysis revealed taxa-specific expression patterns. Some highly conserved OGs exhibited stable protein expression across all species, mainly in essential cellular functions like ribosomal protein synthesis and DNA binding. It seems that bacteria universally invest most of their energy into the expression of housekeeping genes, which are essential for growth. Nevertheless, expression levels of ribosomal proteins depend on the growth phase and the growth rate (71, 72). Conversely, taxa-specific expression patterns emerged for more specialized proteins such as the cysteine desulfurases, which are crucial for iron-sulfur (Fe-S) cluster formation in respiration, metabolism, and gene regulation (73). Notably, distinct expression profiles were observed for LcsS and Csd, group I (consensus motif SSGSACTS) and group II (consensus motif RXGHHCA) type desulfurases (73), respectively, across Pseudomonadota and Bacillota, suggesting diverse phyla-specific sulfur activation strategies.

It is important to note that all bacteria were cultured under one specified condition (Supplemental Table S2), providing a snapshot of protein expression and reflecting the molecular phenotype. Broader sampling under varied conditions will further elucidate expressible proteomes and enhance our understanding of adaptations and protein functions, as demonstrated in model organisms like *E. coli* (74) or *P. aeruginosa* (75). We have established the infrastructure to incorporate such datasets into ProteomicsDB in the future.

Bacterial identification is pivotal in infectious disease diagnosis, food-borne disease outbreaks, water quality control, and antibiotic resistance testing. While 16S rRNA gene sequencing and MALDI-TOF biotyping are currently the most commonly applied methods, they exhibit limited taxonomic resolution, particularly for closely related taxa, and sometimes depending on the taxonomic group. MS-based proteomics offers enhanced discrimination by focusing on unique peptides (14, 18) or considering all detected peptides with stringent identification scoring (20, 51, 76, 77).

MS2Bac, the proteomics-based bacterial identification approach presented here, achieved >99% species-level and >89% strain-level accuracy across 303 species and two strain-level datasets used as benchmarks. We note that the diversity dataset is biased towards type strains and strains with a sequenced genome. Nevertheless, MS2Bac outperformed MALDI-TOF and FTIR in food-derived samples and correlated well with 16S rRNA gene sequencing (94%). We detected diverse bacterial species and antimicrobial resistance markers in a large clinical sample set. We found at least one beta-lactamase expressed in every fourth clinical isolate, including many ESBLs, highlighting the urgent need for more antimicrobial resistance monitoring.

Effective proteomics-based identification with MS2Bac relies on an accurate and comprehensive reference database, ideally encompassing all bacterial species and strains to fully capture genomic diversity. MS2Bac identifies the species with the highest number of mapped PSMs as the detected species. When the correct species is represented in the reference database, accurate identification is achieved for over 99% of the bacterial species analyzed. This has been demonstrated in the present study using an exceptionally diverse set of 303 bacterial species. However, if the bacterial species being investigated is not represented in the reference database (as this was in our study the case for *B. cereus*), MS2Bac will classify it to the closest relative (in our study, this was *B. thuringiensis* (Fig. 3*D*). Although the number of PSM counts will vary depending on the size and composition of the reference database, the correct species should consistently rank first, as tryptic peptidomes are highly distinct between species (Supplemental Figs. S5 and S6). Moreover, the reference database used by MS2Bac can be regularly updated, given that new bacterial genomes are continuously added to public repositories.

MS2Bac's discriminative power strongly depends on the taxonomic relationship within the reference database—the closer related species and strains, the fewer unique peptides will be available for separating the best from the second-best hit. To address this issue, MS2Bac performs analysis in two iterations: the first focuses on species-level identification, and the second includes strain-level information only for the identified genus. Iterative searching was essential for strain-level typing in this study and has been proven effective in two other studies (20, 51). Hence, it is the most promising strategy for bacterial identification at the subspecies level. Notably, the continuously growing knowledge in databases like NCBI (3) and UniProt (50) can seamlessly be implemented into MS2Bac by updating the reference database.

Although bottom-up proteomics holds great potential for strain-resolved bacterial identification in clinics, the biggest challenge is its turnaround time. So far, MS2Bac has been tested only on pure cultures, requiring lengthy preparation and analysis times. Yet, we have demonstrated that with a shortened digestion time and faster data acquisition, MS2Bac's identification accuracy was still maintained. Other technical advancements like direct infusion (51), handheld MS probe (78), automation, and faster search algorithms (79), or even de-novo search algorithms (80) promise to further accelerate MS-based bacterial identification. Bottom-up proteomics also shows great potential for investigating mixed cultures (19) and identifying bacteria directly from surgical specimens (78), urine (18), or blood (81), bypassing the need for cultivation. Future research will explore MS2Bac's potential specifically for mixed sample types. Other limiting factors are the comparatively high costs and the need for specialized personnel. Assuming that these factors can be resolved, we expect that MS-based proteomic methods will become part of the clinical routine due to their superior resolution.

In conclusion, our study establishes a broad proteomic resource that details protein expression profiles across an unprecedentedly wide taxonomic range of over 300 different bacterial species. This resource facilitated the development of a new bacterial identification algorithm with numerous applications in clinics, the food and cosmetic industry, environmental studies, and epidemiological investigations. In the food industry, bottom-up proteomics is an attractive method due to its high resolving power and the independence from spectral libraries. It can be used for identifying contaminant or spoilage bacteria, tracing contamination routes, or validating the identity of starter cultures. Additionally, proteomics data can aid in differentiating bacterial strains, identifying virulence factors, and detecting toxins. This is particularly relevant in clinical settings, where antibiotic-resistance markers can be detected in an all-in-one tool (10). Furthermore, high-resolution identification may allow for the differentiation of clinically relevant serovars, although further investigations are needed. Finally, environmental studies can benefit from the taxonomically diverse reference database used within MS2Bac, which includes less-commonly studied strains not found in standard spectral libraries for MALDI-TOF or FTIR.

## Data availability

All the mass spectrometry proteomics data has been deposited to the ProteomeXchange Consortium (https://massive.ucsd.edu/ProteoSAFe/static/massive.jsp) *via* the MassIVE partner repository (82) with the dataset identifier ftp://MSV000096603@massive.ucsd.edu. An SDRF (83) file, a summary of reference database fasta files, the four reference fasta files, a ProteomicsDB output file including rescored peptide and protein identifications, MS2Bac identification results, orthogroups, functional annotations, and all Source data files have also been deposited on MassIVE under the same link. The source code for the MS2Bac algorithm and a readme file with installation and usage instructions (and the reference database fasta files are uploaded to Zenodo and) can be downloaded *via* Zenodo using the following link: https://zenodo.org/records/14419526.

A readme file is provided with installation and usage instructions.

## Supplemental data

This article contains supplemental data (62, 67, 83).

## Conflicts of Interests

B. K. and M. Wi. are founders and shareholders of MSAID GmbH and OmicScouts GmbH. They have no operational role in either of the two companies. All other authors declare no competing interests.

## References

[1] Bahram M., Netherway T., Frioux C., Ferretti P., Coelho L.P., Geisen S. (2021). Metagenomic assessment of the global diversity and distribution of bacteria and fungi. Environ. Microbiol..

[2] Nayfach S., Roux S., Seshadri R., Udwary D., Varghese N., Schulz F. (2021). A genomic catalog of Earth's microbiomes. Nat. Biotechnol..

[3] Sayers E.W., Beck J., Bolton E.E., Brister J.R., Chan J., Comeau D.C. (2024). Database resources of the national center for Biotechnology information. Nucleic Acids Res..

[4] Aebersold R., Mann M. (2016). Mass-spectrometric exploration of proteome structure and function. Nature.

[5] Gouveia D., Grenga L., Pible O., Armengaud J. (2020). Quick microbial molecular phenotyping by differential shotgun proteomics. Environ. Microbiol..

[6] Muller J.B., Geyer P.E., Colaço A.R., Treit P.V., Strauss M.T., Oroshi M. (2020). The proteome landscape of the kingdoms of life. Nature.

[7] Abele M., Doll E., Bayer F.P., Meng C., Lomp N., Neuhaus K. (2023). Unified workflow for the rapid and in-depth characterization of bacterial proteomes. Mol. Cell Proteomics.

[8] Samaras P., Schmidt T., Frejno M., Gessulat S., Reinecke M., Jarzab A. (2020). ProteomicsDB: a multi-omics and multi-organism resource for life science research. Nucleic Acids Res..

[9] Wenning M., Breitenwieser F., Konrad R., Huber I., Busch U., Scherer S. (2014). Identification and differentiation of food-related bacteria: a comparison of FTIR spectroscopy and MALDI-TOF mass spectrometry. J. Microbiol. Methods.

[10] Blumenscheit C., Pfeifer Y., Werner G., John C., Schneider A., Lasch P., Doellinger J. (2021). Unbiased antimicrobial resistance detection from clinical bacterial isolates using proteomics. Anal. Chem..

[11] Alves G., Ogurtsov A., Karlsson R., Jaén-Luchoro D., Piñeiro-Iglesias B., Salvà-Serra F. (2022). Identification of antibiotic resistance proteins *via* MiCId's augmented workflow. A mass spectrometry-based proteomics approach. J. Am. Soc. Mass Spectrom..

[12] Karlsson R., Davidson M., Svensson-Stadler L., Karlsson A., Olesen K., Carlsohn E., Moore E.R.B. (2012). Strain-level typing and identification of bacteria using mass spectrometry-based proteomics. J. Proteome Res..

[13] Lasch P., Schneider A., Blumenscheit C., Doellinger J. (2020). Identification of Microorganisms by liquid chromatography-mass spectrometry (LC-MS(1)) and in silico peptide mass libraries. Mol. Cell Proteomics.

[14] Karlsson R., Thorsell A., Gomila M., Salvà-Serra F., Jakobsson H.E., Gonzales-Siles L. (2020). Discovery of species-unique peptide biomarkers of bacterial pathogens by tandem mass spectrometry-based proteotyping. Mol. Cell Proteomics.

[15] Russo R., Valletta M., Rega C., Marasco R., Muscariello L., Pedone P.V. (2019). Reliable identification of lactic acid bacteria by targeted and untargeted high-resolution tandem mass spectrometry. Food Chem..

[16] Berendsen E.M., Levin E., Braakman R., Prodan A., van Leeuwen H.C., Paauw A. (2020). Untargeted accurate identification of highly pathogenic bacteria directly from blood culture flasks. Int. J. Med. Microbiol..

[17] Boulund F., Karlsson R., Gonzales-Siles L., Johnning A., Karami N., Al-Bayati O. (2017). Typing and characterization of bacteria using bottom-up tandem mass spectrometry proteomics. Mol. Cell Proteomics.

[18] Roux-Dalvai F., Gotti C., Leclercq M., Hélie M.C., Boissinot M., Arrey T.N. (2019). Fast and accurate bacterial species identification in urine specimens using LC-MS/MS mass spectrometry and machine learning. Mol. Cell Proteomics.

[19] Alves G., Wang G., Ogurtsov A.Y., Drake S.K., Gucek M., Sacks D.B. (2018). Rapid classification and identification of multiple Microorganisms with accurate statistical significance *via* high-resolution tandem mass spectrometry. J. Am. Soc. Mass Spectrom..

[20] Kuhring M., Doellinger J., Nitsche A., Muth T., Renard B.Y. (2020). TaxIt: an iterative computational pipeline for untargeted strain-level identification using MS/MS spectra from pathogenic single-organism samples. J. Proteome Res..

[21] Dworzanski J.P., Deshpande S.V., Chen R., Jabbour R.E., Snyder A.P., Wick C.H. (2006). Mass spectrometry-based proteomics combined with bioinformatic tools for bacterial classification. J. Proteome Res..

[22] Deutschland B.d.I.u.H. (2015). Einstufung von Prokaryonten (Bacteria und Archaea) in Risikogruppen. Gemeinsames Ministerialblatt.

[23] Doellinger J., Schneider A., Hoeller M., Lasch P. (2020). Sample preparation by easy extraction and digestion (SPEED) - a universal, rapid, and detergent-free protocol for proteomics based on acid extraction. Mol. Cell Proteomics.

[24] Rappsilber J., Mann M., Ishihama Y. (2007). Protocol for micro-purification, enrichment, pre-fractionation and storage of peptides for proteomics using StageTips. Nat. Protoc..

[25] Zolg D.P., Wilhelm M., Yu P., Knaute T., Zerweck J., Wenschuh H. (2017). PROCAL: a set of 40 peptide standards for retention time indexing, column performance monitoring, and collision energy calibration. Proteomics.

[26] Cox J., Neuhauser N., Michalski A., Scheltema R.A., Olsen J.V., Mann M. (2011). Andromeda: a peptide search engine integrated into the MaxQuant environment. J. Proteome Res..

[27] Tyanova S., Temu T., Cox J. (2016). The MaxQuant computational platform for mass spectrometry-based shotgun proteomics. Nat. Protoc..

[28] Picciani M., Gabriel W., Giurcoiu V.G., Shouman O., Hamood F., Lautenbacher L. (2023). Oktoberfest: open-source spectral library generation and rescoring pipeline based on Prosit. Proteomics.

[29] Wilhelm M., Zolg D.P., Graber M., Gessulat S., Schmidt T., Schnatbaum K. (2021). Deep learning boosts sensitivity of mass spectrometry-based immunopeptidomics. Nat. Commun..

[30] Gessulat S., Schmidt T., Zolg D.P., Samaras P., Schnatbaum K., Zerweck J. (2019). Prosit: proteome-wide prediction of peptide tandem mass spectra by deep learning. Nat. Methods.

[31] The M., MacCoss M.J., Noble W.S., Käll L. (2016). Fast and accurate protein false discovery rates on large-scale proteomics data sets with percolator 3.0. J. Am. Soc. Mass Spectrom..

[32] The M., Samaras P., Kuster B., Wilhelm M. (2022). Reanalysis of ProteomicsDB using an accurate, sensitive, and scalable false discovery rate estimation approach for protein groups. Mol. Cell Proteomics.

[33] Schwanhausser B., Busse D., Li N., Dittmar G., Schuchhardt J., Wolf J. (2011). Global quantification of mammalian gene expression control. Nature.

[34] Tyanova S., Temu T., Sinitcyn P., Carlson A., Hein M.Y., Geiger T. (2016). The Perseus computational platform for comprehensive analysis of (prote)omics data. Nat. Methods.

[35] Cox J., Hein M.Y., Luber C.A., Paron I., Nagaraj N., Mann M. (2014). Accurate proteome-wide label-free quantification by delayed normalization and maximal peptide ratio extraction, termed MaxLFQ. Mol. Cell Proteomics.

[36] Hunter J.D. (2007). Matplotlib: a 2D graphics environment. Comput. Sci. Eng..

[37] Waskom M.L. (2021). seaborn: statistical data visualization. J. Open Source Softw..

[38] Alcock B.P., Huynh W., Chalil R., Smith K.W., Raphenya A.R., Wlodarski M.A. (2023). Card 2023: expanded curation, support for machine learning, and resistome prediction at the Comprehensive Antibiotic Resistance Database. Nucleic Acids Res..

[39] Yoon S.H., Ha S.M., Kwon S., Lim J., Kim Y., Seo H. (2017). Introducing EzBioCloud: a taxonomically united database of 16S rRNA gene sequences and whole-genome assemblies. Int. J. Syst. Evol. Microbiol..

[40] McGinnis S., Madden T.L. (2004). BLAST: at the core of a powerful and diverse set of sequence analysis tools. Nucleic Acids Res..

[41] Lauterbach A., Usbeck J.C., Behr J., Vogel R.F. (2017). MALDI-TOF MS typing enables the classification of brewing yeasts of the genus Saccharomyces to major beer styles. PLoS One.

[42] Schoch C.L., Ciufo S., Domrachev M., Hotton C.L., Kannan S., Khovanskaya R. (2020). NCBI Taxonomy: a comprehensive update on curation, resources and tools. Database (Oxford).

[43] Letunic I., Bork P. (2021). Interactive Tree of Life (iTOL) v5: an online tool for phylogenetic tree display and annotation. Nucleic Acids Res..

[44] Emms D.M., Kelly S. (2019). OrthoFinder: phylogenetic orthology inference for comparative genomics. Genome Biol..

[45] Paysan-Lafosse T., Blum M., Chuguransky S., Grego T., Pinto B.L., Salazar G.A. (2023). InterPro in 2022. Nucleic Acids Res..

[46] Toronen P., Medlar A., Holm L. (2018). PANNZER2: a rapid functional annotation web server. Nucleic Acids Res..

[47] Ashburner M., Ball C.A., Blake J.A., Botstein D., Butler H., Cherry J.M. (2000). Gene ontology: tool for the unification of biology. The Gene Ontology Consortium. Nat. Genet..

[48] Gene Ontology C., Aleksander S.A., Balhoff J., Carbon S., Cherry J.M., Drabkin H.J. (2023). The gene ontology knowledgebase in 2023. Genetics.

[49] Klopfenstein D.V., Zhang L., Pedersen B.S., Ramírez F., Warwick Vesztrocy A., Naldi A. (2018). GOATOOLS: a Python library for Gene Ontology analyses. Sci. Rep..

[50] UniProt C. (2023). UniProt: the universal protein knowledgebase in 2023. Nucleic Acids Res..

[51] Chabas M., Gaillard J.C., Alpha-Bazin B., Armengaud J. (2024). Flash MS/MS proteotyping allows identifying microbial isolates in 36 s of mass spectrometry signal. Proteomics.

[52] Kong A.T., Leprevost F.V., Avtonomov D.M., Mellacheruvu D., Nesvizhskii A.I. (2017). MSFragger: ultrafast and comprehensive peptide identification in mass spectrometry-based proteomics. Nat. Methods.

[53] Ciufo S., Kannan S., Sharma S., Badretdin A., Clark K., Turner S. (2018). Using average nucleotide identity to improve taxonomic assignments in prokaryotic genomes at the NCBI. Int. J. Syst. Evol. Microbiol..

[54] Huerta-Cepas J., Dopazo J., Gabaldon T. (2010). ETE: a python environment for tree exploration. BMC Bioinformatics.

[55] Lan R., Reeves P.R. (2002). Escherichia coli in disguise: molecular origins of Shigella. Microbes Infect..

[56] Young J.M., Kuykendall L.D., Martínez-Romero E., Kerr A., Sawada H. (2003). Classification and nomenclature of Agrobacterium and Rhizobium. Int. J. Syst. Evol. Microbiol..

[57] Reimer L.C., Sardà Carbasse J., Koblitz J., Ebeling C., Podstawka A., Overmann J. (2022). BacDive in 2022: the knowledge base for standardized bacterial and archaeal data. Nucleic Acids Res..

[58] Goloborodko A.A., Levitsky L.I., Ivanov M.V., Gorshkov M.V. (2013). Pyteomics--a Python framework for exploratory data analysis and rapid software prototyping in proteomics. J. Am. Soc. Mass Spectrom..

[59] McKinney W. (2010). Data structures for statistical computing in python. Proc. 9th Python Sci. Conf..

[60] Harris C.R., Millman K.J., van der Walt S.J., Gommers R., Virtanen P., Cournapeau D. (2020). Array programming with NumPy. Nature.

[61] Deutsch E.W. (2010). The PeptideAtlas project. Methods Mol. Biol..

[62] Huang Q., Szklarczyk D., Wang M., Simonovic M., von Mering C. (2023). PaxDb 5.0: curated protein quantification data suggests adaptive proteome changes in yeasts. Mol. Cell Proteomics.

[63] Baur H., Luethi E., Stalon V., Mercenier A., Haas D. (1989). Sequence analysis and expression of the arginine-deiminase and carbamate-kinase genes of Pseudomonas aeruginosa. Eur. J. Biochem..

[64] Verhoogt H.J., Smit H., Abee T., Gamper M., Driessen A.J., Haas D. (1992). arcD, the first gene of the arc operon for anaerobic arginine catabolism in Pseudomonas aeruginosa, encodes an arginine-ornithine exchanger. J. Bacteriol..

[65] Keller A.N., Yang X., Wiedermannová J., Delumeau O., Krásný L., Lewis P.J. (2014). epsilon, a new subunit of RNA polymerase found in gram-positive bacteria. J. Bacteriol..

[66] Meldal B.H.M., Perfetto L., Combe C., Lubiana T., Ferreira Cavalcante J.V., Bye-A-Jee H. (2022). Complex Portal 2022: new curation frontiers. Nucleic Acids Res..

[67] Elias J.E., Gygi S.P. (2010). Target-decoy search strategy for mass spectrometry-based proteomics. Methods Mol. Biol..

[68] Brenner D.J., Fanning G.R., Steigerwalt A.G., Orskov I., Orskov F. (1972). Polynucleotide sequence relatedness among three groups of pathogenic Escherichia coli strains. Infect. Immun..

[69] Balazova T., Makovcová J., Šedo O., Slaný M., Faldyna M., Zdráhal Z. (2014). The influence of culture conditions on the identification of Mycobacterium species by MALDI-TOF MS profiling. FEMS Microbiol. Lett..

[70] Husna A., Rahman M.M., Badruzzaman A.T.M., Sikder M.H., Islam M.R., Rahman M.T. (2023). Extended-spectrum beta-lactamases (ESBL): challenges and opportunities. Biomedicines.

[71] Balakrishnan R., Mori M., Segota I., Zhang Z., Aebersold R., Ludwig C. (2022). Principles of gene regulation quantitatively connect DNA to RNA and proteins in bacteria. Science.

[72] Matamouros S., Gensch T., Cerff M., Sachs C.C., Abdollahzadeh I., Hendriks J. (2023). Growth-rate dependency of ribosome abundance and translation elongation rate in Corynebacterium glutamicum differs from that in Escherichia coli. Nat. Commun..

[73] Ayala-Castro C., Saini A., Outten F.W. (2008). Fe-S cluster assembly pathways in bacteria. Microbiol. Mol. Biol. Rev..

[74] Mori M., Zhang Z., Banaei-Esfahani A., Lalanne J.B., Okano H., Collins B.C. (2021). From coarse to fine: the absolute Escherichia coli proteome under diverse growth conditions. Mol. Syst. Biol..

[75] Reales-Calderon J.A., Sun Z., Mascaraque V., Pérez-Navarro E., Vialás V., Deutsch E.W. (2021). A wide-ranging Pseudomonas aeruginosa PeptideAtlas build: a useful proteomic resource for a versatile pathogen. J. Proteomics.

[76] Penzlin A., Lindner M.S., Doellinger J., Dabrowski P.W., Nitsche A., Renard B.Y. (2014). Pipasic: similarity and expression correction for strain-level identification and quantification in metaproteomics. Bioinformatics.

[77] Pible O., Allain F., Jouffret V., Culotta K., Miotello G., Armengaud J. (2020). Estimating relative biomasses of organisms in microbiota using "phylopeptidomics". Microbiome.

[78] Povilaitis S.C., Chakraborty A., Kirkpatrick L.M., Downey R.D., Hauger S.B., Eberlin L.S. (2022). Identifying clinically relevant bacteria directly from culture and clinical samples with a handheld mass spectrometry probe. Clin. Chem..

[79] Lazear M.R. (2023). Sage: an open-source tool for fast proteomics searching and quantification at scale. J. Proteome Res..

[80] Eloff K., Kalogeropoulos K., Morell O., Mabona A., Jespersen J.B., Williams W. (2023). *De novo* peptide sequencing with InstaNovo: accurate, database-free peptide identification for large scale proteomics experiments. bioRxiv.

[81] Kondori N., Kurtovic A., Piñeiro-Iglesias B., Salvà-Serra F., Jaén-Luchoro D., Andersson B. (2021). Mass spectrometry proteotyping-based detection and identification of Staphylococcus aureus, Escherichia coli, and Candida albicans in blood. Front. Cell Infect. Microbiol.

[82] Deutsch E.W., Bandeira N., Sharma V., Perez-Riverol Y., Carver J.J., Kundu D.J. (2019). The ProteomeXchange consortium in 2020: enabling ‘big data’ approaches in proteomics. Nucleic Acids Res..

[83] Claeys T., Van Den Bossche T., Perez-Riverol Y., Gevaert K., Vizcaíno J.A., Martens L. (2023). lesSDRF is more: maximizing the value of proteomics data through streamlined metadata annotation. Nat. Commun..

