# Supplementary material for: Proteomic Diversity in Bacteria: Insights and Implications for Bacterial Identification
Source: Mol Cell Proteomics. 2025 Jan 27;24(3):100917. doi: 10.1016/j.mcpro.2025.100917 (PMC11919601; doi:10.1016/j.mcpro.2025.100917)
Supplement: Supplemental data [file mmc1.docx]

**Proteomic diversity in bacteria: Insights and implications for bacterial identification**

Miriam Abele^1,2#^, Armin Soleymaniniya^3#^, Florian P. Bayer^2^, Nina Lomp^1^, Etienne Doll^4^, Chen Meng^1^, Klaus Neuhaus^5^, Siegfried Scherer^4^, Mareike Wenning^6^, Nina Wantia^7^, Bernhard Kuster^1,2^, Mathias Wilhelm^3^, Christina Ludwig^1*^

1. Bavarian Center for Biomolecular Mass Spectrometry (BayBioMS), TUM School of Life Sciences, Technical University of Munich, 85354 Freising, Germany
2. Chair of Proteomics and Bioanalytics, TUM School of Life Sciences, Technical University of Munich, 85354 Freising, Germany
3. Computational Mass Spectrometry, TUM School of Life Sciences, Technical University of Munich, 85354 Freising, Germany
4. Research Department Molecular Life Sciences, TUM School of Life Sciences, 85354 Freising, Germany
5. Core Facility Microbiome, ZIEL Institute for Food & Health, Technical University of Munich, 85354 Freising, Germany
6. Bavarian Health and Food Safety Authority, 85764 Oberschleißheim, Germany
7. Institut für Medizinische Mikrobiologie, Immunologie und Hygiene, TUM School of Medicine and Health Department Preclinical Medicine, Technical University of Munich, Munich, Germany

# Contributed equally to this work

* Corresponding author, tina.ludwig@tum.de

**Running title: Proteotyping with MS2Bac**

**Content**

**Supplementary Figures:**

Supplementary Figure S1 - Peptide and protein Identifications across the bacterial domain of life

Supplementary Figure S2 - Visualization in ProteomicsDB

Supplementary Figure S3 – Quantitative exploration of the proteomes across the bacterial domain of life

Supplementary Figure S4 - Exploration of protein expression across bacterial species through the definition of orthogroups and taxon-specific conservation

Supplementary Figure S5 - Unique tryptic peptide sequences in bacteria

Supplementary Figure S6 - Tryptic peptide diversity across the bacterial domain of life

Supplementary Figure S7 - Performance of MS2Bac

Supplementary Figure S8 - : Bacterial sampling at variable cultivation time points leads to differential protein abundances

Supplementary Figure S9 - : Bacterial sampling at variable cultivation conditions leads to differential protein abundances

Supplementary Figure S10 - Strain-level reference databases’ composition

Supplementary Figure S11 - Beta-lactamase detection in clinical isolates

**Supplementary Tables as separate files**

Supplementary Table S1 – Overview of datasets in this study

Supplementary Table S2 – Overview of samples

Supplementary Table S3 – Overview of sample preparation procedures

Supplementary Table S4 – Overview of data acquisition strategies

Supplementary Table S5 – Fasta files used for orthogroup mapping and hamming distance calculation

Supplementary Table S6 – Orthogroups

Supplementary Table S7 – Enrichment analysis orthogroups

Supplementary Table S8 - Fasta files from 13,855 species from the bacterial species-level reference database of MS2Bac

Supplementary Table S9 - Fasta files from 584 strain from the strain-level fungal reference database of MS2Bac

Supplementary Table S10 – Hypothetical protein information

**Source data:**

The mass spectrometry proteomics data has been deposited to the ProteomeXchange Consortium (https://massive.ucsd.edu/ProteoSAFe/static/massive.jsp) via the MassIVE partner repository [1] with the dataset identifier ftp://MSV000096603@massive.ucsd.edu and togetehr with metadata, a ProteomicsDB output file including rescored peptide and protein identifications, MS2Bac identification results, orthogroups, functional annotations, and all Source data files.

SourceData 1a: ProteomicsDB export and metadata

SourceData 1b: Quantified Proteins from ProteomicsDB export and Hamming distance

SourceData 2: Prosit statistics

SourceData 3: Orthogroups statistics

SourceData 4: Number of proteins in ProteomicsDB

SourceData 5: MS2Bac identification results dataset i

SourceData 6: MS2Bac identification results dataset ii

SourceData 7: MS2Bac identification results dataset iii

SourceData 8: MS2Bac identification results dataset iii, second iteration

SourceData 9: MS2Bac identification results dataset iv (Reproducibility)

SourceData 10: MS2Bac identification results dataset v (Pseudomonas)

SourceData 11: MS2Bac second iteration identification results dataset v (Pseudomonas)

SourceData 12: MS2Bac identification results dataset vi (Bacillus)

SourceData 13: MS2Bac second iteration identification results dataset vi (Bacillus)

SourceData 14: MS2Bac identification results dataset vii (Food routine)

SourceData 15: MS2Bac second iteration identification results dataset vii (Food routine)

SourceData 16: MS2Bac identification results dataset viii (Clinical routine)

SourceData 17: MS2Bac second iteration identification results dataset viii – fungi (clinical routine)

**Supplemental Figure 1: Peptide and protein Identifications across the bacterial domain of life**


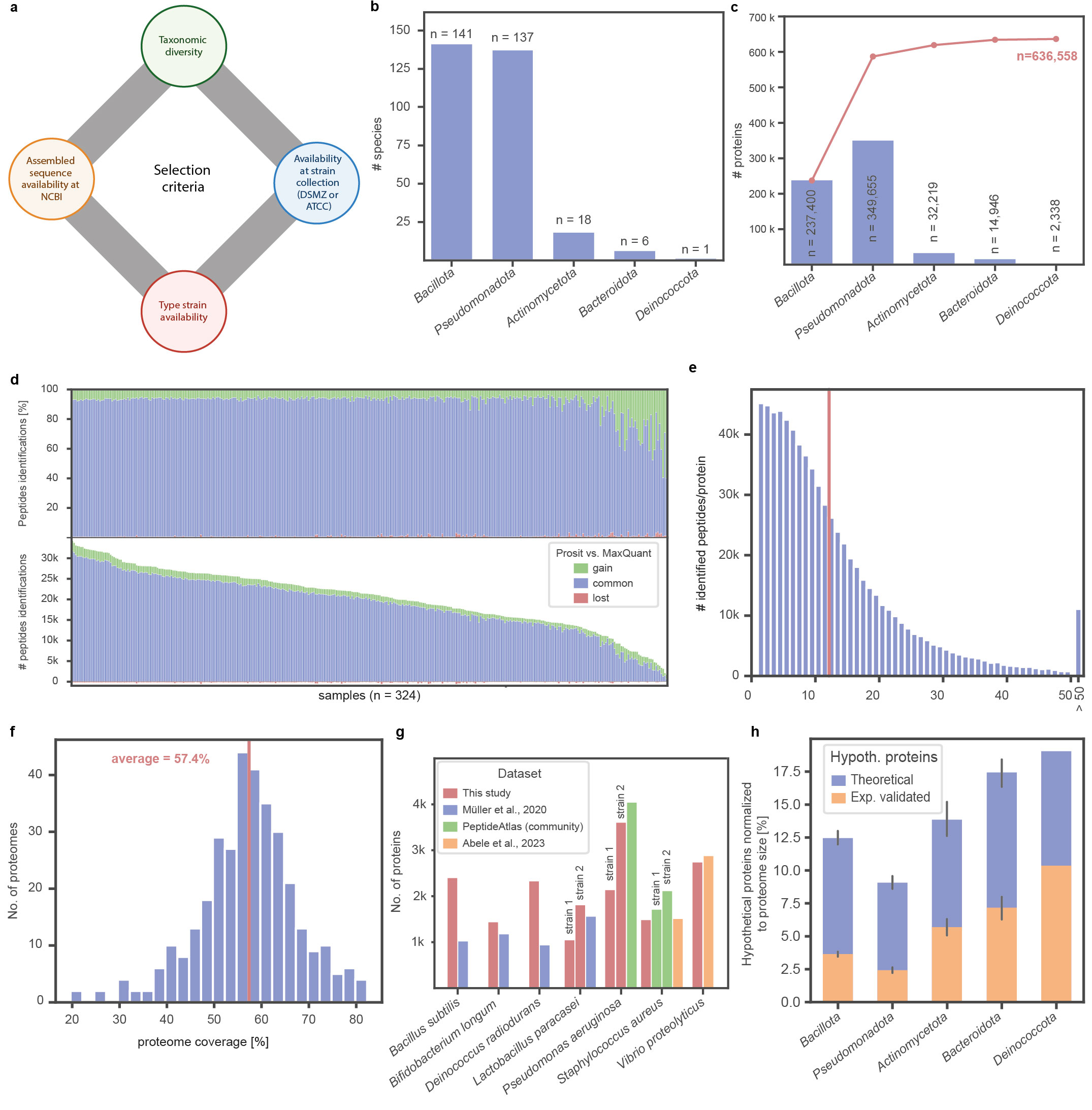


1. Selection criteria for the 303 bacterial species characterized in this dataset.
2. Number of species characterized per phylum.
3. Total number of detected proteins per phylum (bar) and cumulative number of detected proteins across phyla (red line).
4. The effect of performing a rescoring analysis step with Prosit on the number of confidentially identified peptides. Peptides only identified with 100% MaxQuant + Prosit + Percolator but not in 100% MaxQuant + Percolator are shown in green, peptides identified in both are shown in blue, and peptides not identified with Prosit are shown in red. The upper panel shows relative numbers, and the lower panel shows absolute peptide numbers at 1% peptide FDR.
5. Overview of identified peptides per protein across all samples.
6. Fraction of hypothetical proteins per phylum, as labeled in the NCBI fasta header. The proportion of hypothetical proteins experimentally validated in this study is indicated in orange.
7. Overview of the dataset’s proteome coverages per sample.
8. The number of identified proteins in this study compared to other studies (protein groups) is exemplary for seven species.


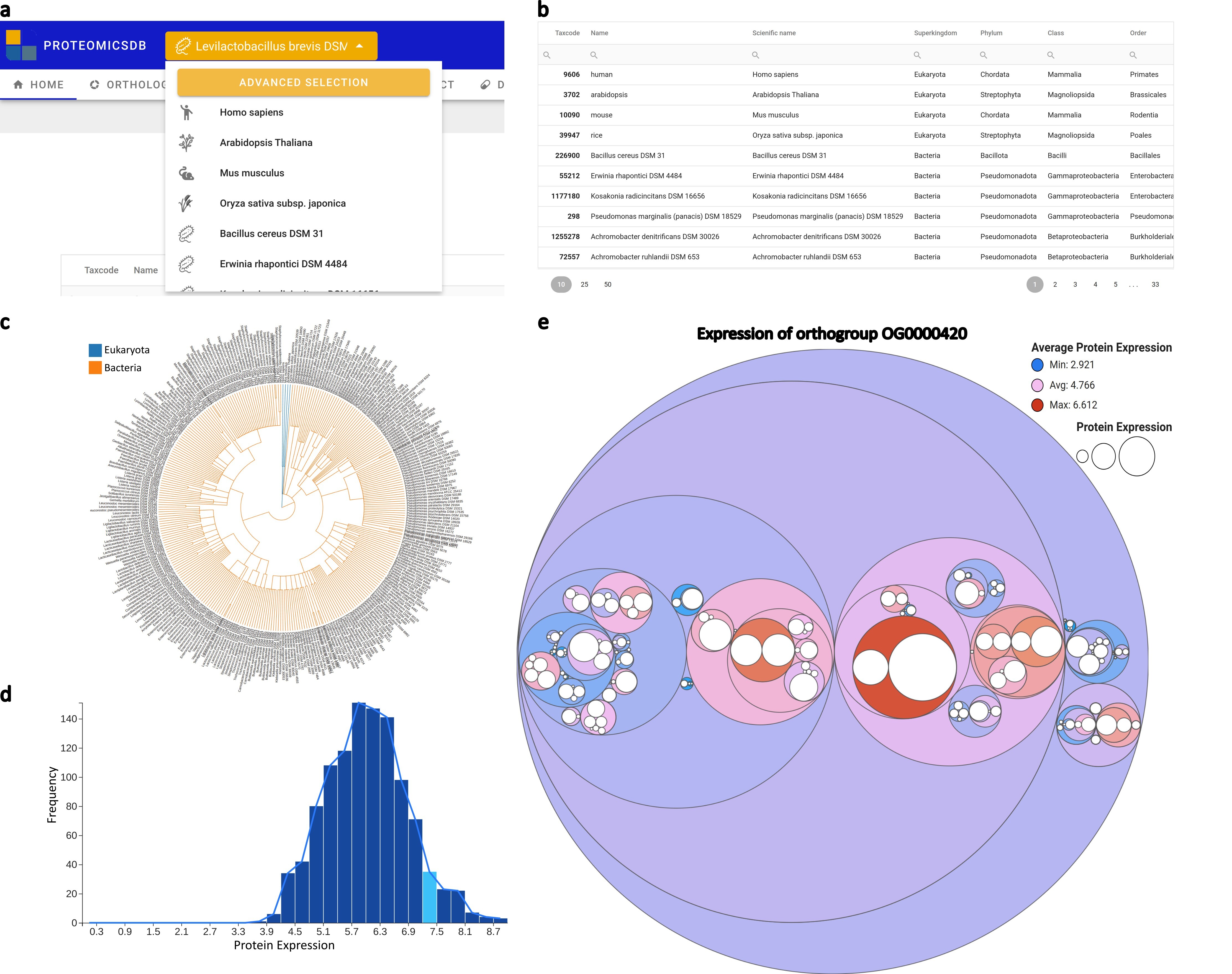


**Supplemental Figure 2: Visualization in ProteomicsDB**

1. Advanced organism selection interface on ProteomicsDB features an autocomplete search function that supports organism exploration through scientific names, tax codes, and collection identifiers.
2. Filterable organism table facilitates streamlined navigation and selection of organisms by enabling users to sort and filter based on desired criteria, like phylogenetic levels.
3. Interactive phylogenetic tree visualization displays all organisms available on ProteomicsDB, allowing users to visually explore and select organisms of interest.
4. Expression landscape visualization for a specific organism replaces traditional anatomograms on ProteomicsDB for single-cell organisms. Shown here is the expression histogram for a polysaccharide deacetylase protein (WP_097546484.1) in *Levilactobacillus brevis* DSM 20556. The highlighted light blue bin represents the abundance level of the specified protein. Expression values are log_10_-transformed riBAQ values shifted positively by 10 units (see Methods).
5. Bubble plot representation of the abundance estimates of all proteins associated with a single orthogroup (here OG0000420; polysaccharide deacetylase family proteins) across various strains. Each white circle corresponds to a protein, with circle size representing abundance (small: low abundance; large: high abundance). Protein abundances are z-scored and shifted positively by 5 units (see Methods). Outer circles represent increasing phylogenetic levels, from strain (innermost) to species, genus, family, order, class, phylum, and super kingdom (outermost). Background color of each circle reflects the average protein abundance at that phylogenetic level, ranging from low (blue) to average (pink) to high (red). The interactive visualization on ProteomicsDB allows zooming and navigation by clicking on specific circles.


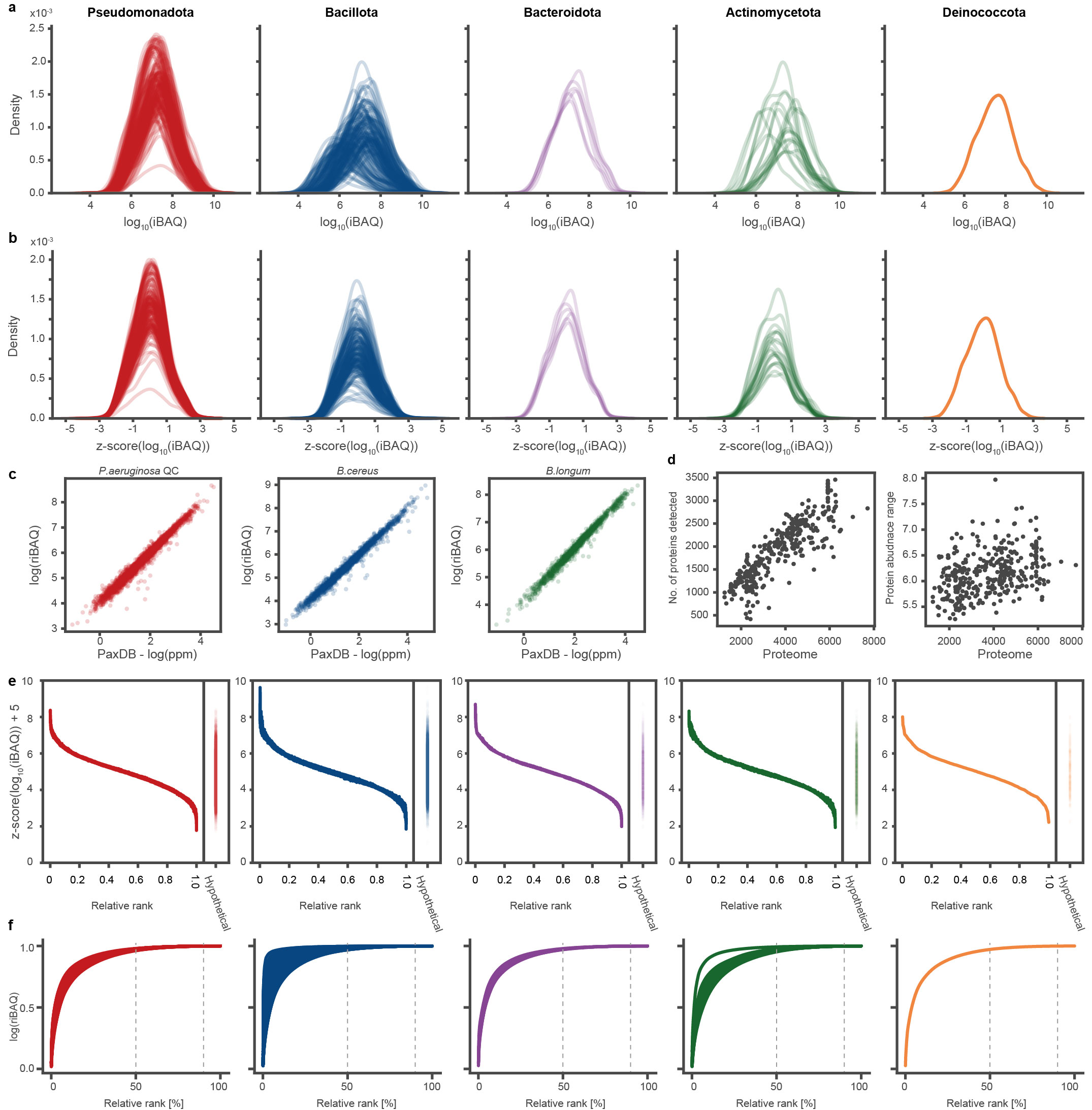


**Supplemental Figure 3: Quantitative exploration of the proteomes across the bacterial domain of life**

1. Distribution plots of log-transformed intensity-based absolute protein quantities (iBAQs) of each species determined by mass-spectrometry-based proteomics. Samples are separated according to phylum. Red: Pseudomonadota, blue: Bacillota, purple: *Bacteroidota*, green: Actinomycetota, orange: *Deinococcota*.
2. Same as (a) but for z-scored and log10-transformed iBAQs.
3. Correlation between log-transformed iBAQs and absolute protein abundances (ppm) computed by PaxDB [2], a database for absolute protein abundances (https://www.pax-db.org/), using the same measurement files as input.
4. In our dataset, proteome size and number of detected proteins show a decent correlation (left panel), while proteome size and protein abundance range are not correlated (right panel).
5. Each bacterium's protein abundance range is separated according to phylum. The color code is the same as in (a). The abundances of all hypothetical (as described in the NCBI fasta headers) are highlighted in the right panel of each phylum-specific plot.
6. Cumulative log-transformed relative iBAQs plotted against their relative rank within each bacterium and separated according to phylum. It has the same color code as (a).


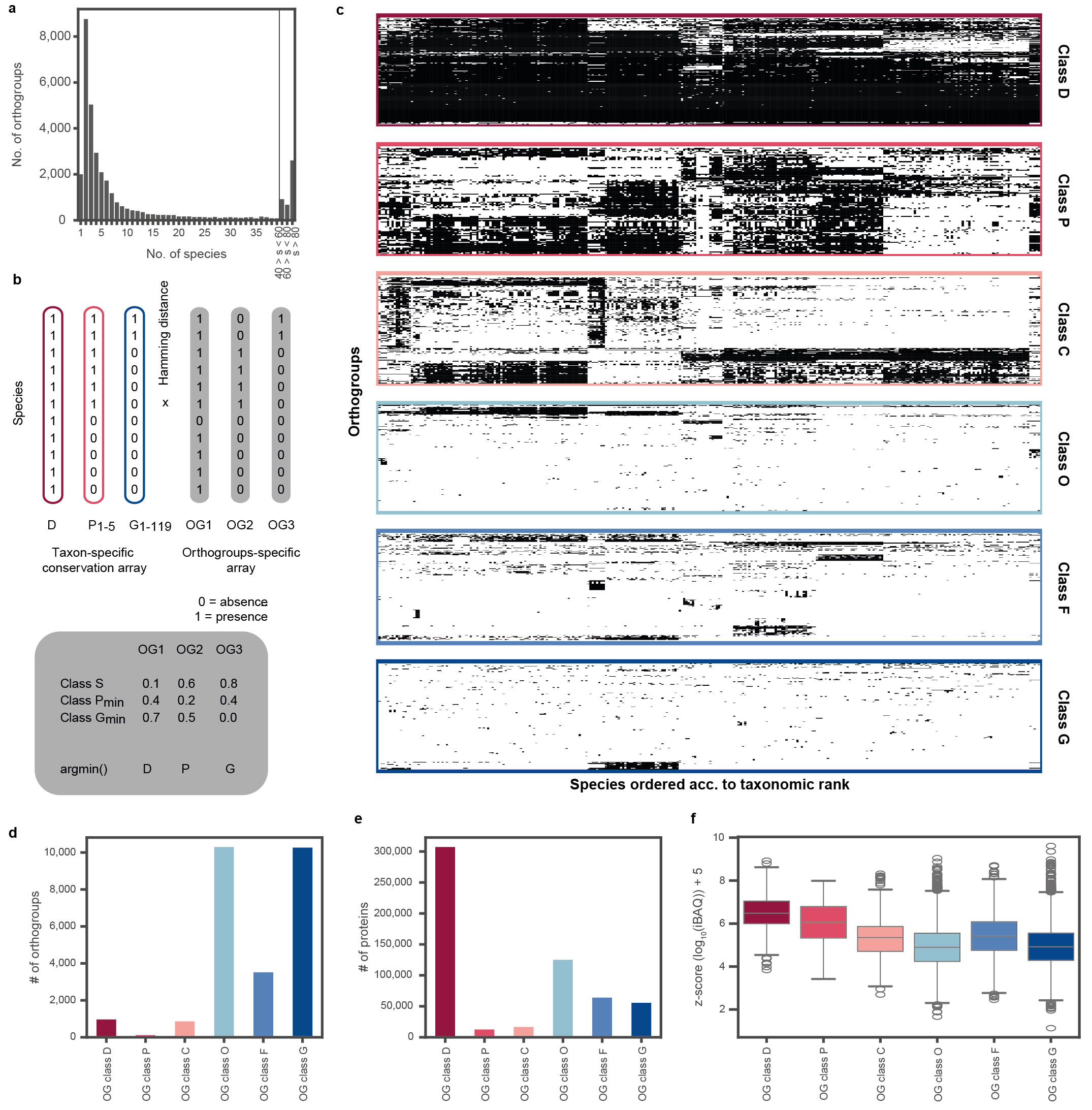


**Supplemental Figure 4: Exploration of protein expression across bacterial species through the definition of orthogroups and taxon-specific conservation**

1. Histogram visualizing the number of orthogroups (OGs) across bacterial species.
2. Schematic illustration of OG classification according to taxon-specific conservation. First, a binary, taxon-specific conservation array (TSCA), using ‘1s’ (OG is expected to be present in the proteome) and ‘0s’ (OG is expected to be absent in the proteome) are defined. They reflect the phylogenetic conservation between species. A TSCA of an OG conserved across all bacterial species consists of only ’1s’. In contrast, TSCAs from OGs conserved only across one taxon consist of ‘1s’ in all species from this taxon and ‘0s’ in all others. Second, we defined OG-specific arrays (OGAs), in which a ‘1’ indicates that at least one protein from the respective OG was found in the reference proteome. In contrast, ‘0s’ indicate no protein exists for this OG in the respective species. Finally, similarities between TSCAs and OGAs were compared using the hamming distance, which allowed an OG classification according to taxonomic conservation into six taxons: domain (D), phylum (P), class (C), order (O), family (F), and genus (G).
3. Binary heatmap of OGAs for each of the six conservation classes. Displayed are the presence (black) or absence (white) of each OG across the characterized proteomes (NCBI fasta files) of the bacterial domain of life. Species on the x-axis were ordered according to their taxonomic relation. A maximum of 100 random examples are shown per class.
4. Number of quantified OGs per conservation class.
5. Number of quantified proteins per conservation class.
6. Boxplots of each conservation class were plotted with the median z-scored log_10_(iBAQ) + 5 of paralogues and the maximal z-scored log_10_(iBAQ) + 5 of homologs per quantified orthogroup.


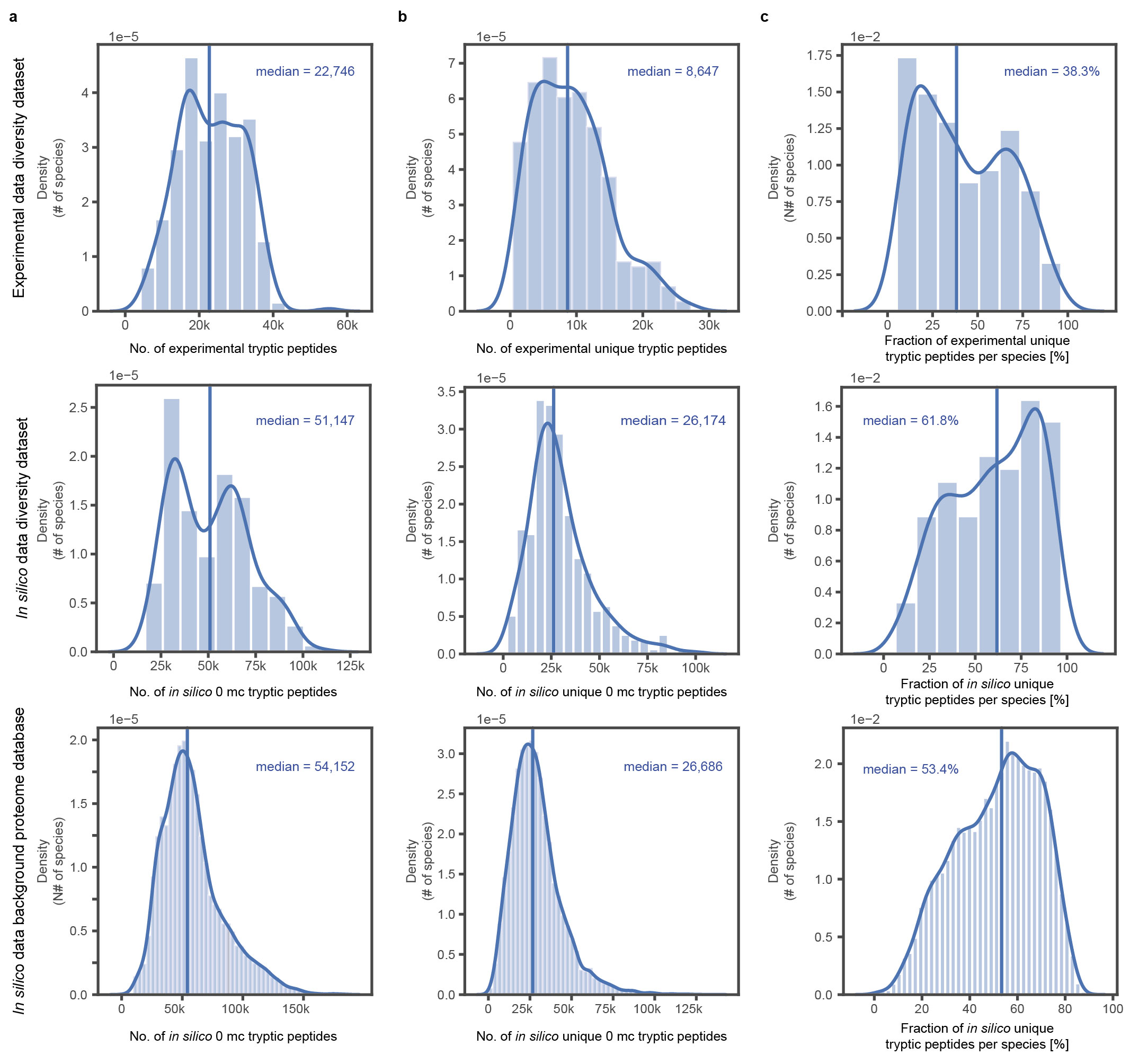


**Supplemental Figure 5: Unique tryptic peptide sequences in bacteria**

1. Histogram of identified peptides across strains. Upper panel: All experimentally identified tryptic peptides (up to two missed cleavages (mc) as reported in ProteomicsDB) in the 14 SPD dataset from this study. Replicate results were concatenated to one results file (n = 318 strains). Middle panel: Peptides with zero missed cleavages from an *in silico* digested of all strain-level proteomes in the 14 SPD dataset (n = 318 strains). Lower panel: Peptides with zero missed cleavages from an *in silico* digested of all proteomes in proteomes from NCBI, which were later included in the species-level reference database (n = 13,855 species).
2. Peptides mapped exclusively to one reference proteome were annotated as unique peptides. Unique peptides can be present twice in the same bacterium. Panel ordering as described above.
3. Fractions of unique tryptic peptides per species were calculated for all experimentally detected peptides (upper panel), all *in silico* digested peptides (middle panel), and all *in silico* digested peptides entailied in the species-level reference database from NCBI (lower panel).


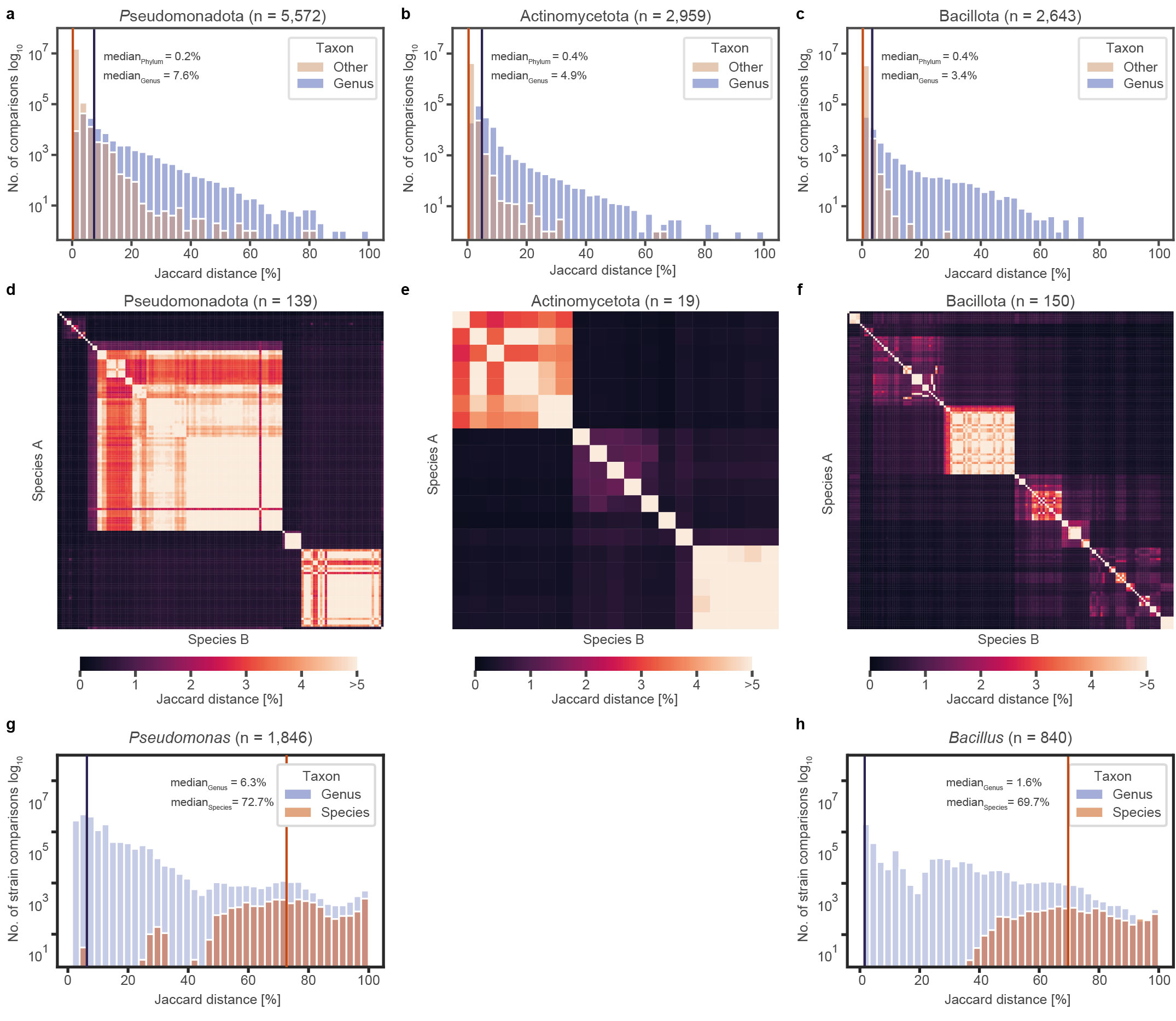


**Supplemental Figure 6: Tryptic peptide diversity across the bacterial domain of life**

1. Pairwise comparison of theoretical tryptic peptidomes among species from the phylum Pseudomonadota (peptide length constraint 7-30 amino acids, maximal number of missed cleavages = 0, string comparisons). Considering all sequenced Pseudomonadota species deposited to NCBI (06 March 2023), only one representative strain per species was selected, resulting in 5,572 Pseudomonadota species. The Jaccard distance was used as a measure for tryptic peptidome similarity. Shortly, the intersection of tryptic peptide sequences was divided by the two species' union of tryptic peptide sequences. Blue: Jaccard distance of peptidomes that belong to the same genus; brown: Jaccard distance of peptidomes from all other Pseudomonadota species. The red bar represents the median tryptic peptidome similarity over all Pseudomonadota species.
2. Same as (a), but for the phylum Actinomycetota, including 2,959 species from NCBI.
3. Same as (a), but for the phylum Bacillota, including 2,643 species from NCBI.
4. Heatmap of pairwise theoretical tryptic peptide overlap normalized to the union of two species (=Jaccard distance) of 139 Pseudomonadota strains analyzed in the 14 samples per day (SPD) diversity dataset. Species are sorted according to NCBI’s taxonomy.
5. Same as (d), but for the phylum Actinomycetota, including 19 strains analyzed in the 14 SPD data set.
6. Same as (d), but for the phylum Bacillota, including 150 strains analyzed in the 14 SPD data set.
7. Same as (a), but for strains of the genus *Pseudomonas*. A maximum of ten strains per species were selected.
8. Same as (g), but for strains of the genus *Bacillus*.


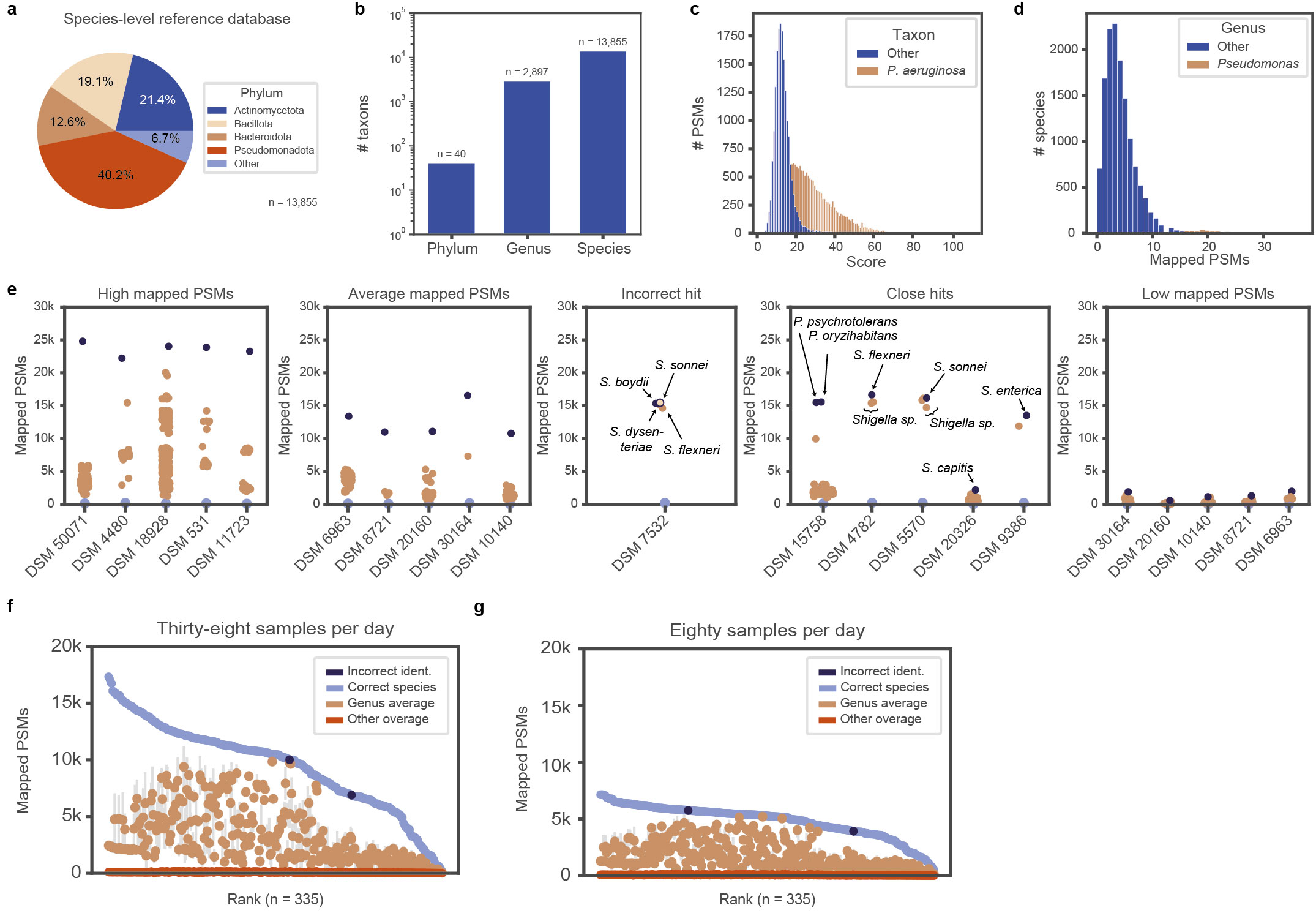


**Supplemental Figure 7: Performance of MS2Bac**

1. The pie chart represents the species proportion in MS2Bac’s species-level reference database of 13,855 species downloaded from NCBI.
2. Absolute numbers of MS2Bac’s species-level reference database.
3. Peptide-spectrum matches (PSMs) from a 100% FDR search assigned to the ground truth bacterium (here: *Pseudomonas aeruginosa*) have a higher hyperscore than PSMs assigned to other bacteria. Taxonomically distinct bacteria can be considered “decoy species” in analogy to the well-established target-decoy approach [3] used in LC-MS/MS based proteomics for identifying fragment ion spectra.
4. Histogram of false positive PSMs (defined as PSMs that cannot be assigned to the ground-truth species *Pseudomonas aeruginosa*). False positive PSMs are equally distributed among all non-ground-truth species, with a few more for *Pseudomonas* spp.
5. Examples of samples from bacterial species grouped into five classes: i) samples with high mapped PSM counts; ii) average performing samples; iii) incorrectly classified samples; iv) samples with close first and second hits; v) samples with low mapped PSM counts (from left to right). The dark blue dots represent the correct species, the orange dots represent different species from the same genus, and the light blue dot represents the average number of mapped PSMs over all species belonging to other genera.
6. In the data set acquired at a throughput of 38 samples per day, the number of mapped PSMs follows the phylogenetic relationship of bacteria. The species with the most mapped PSMs (correctly classified in light blue and incorrectly classified in dark blue) is followed by other species of the same genus (average mapped PSMs in orange and standard deviation as grey bar). Species of different genera are accumulating at low mapped PSMs (average PSMs in red and standard deviation as grey bar).
7. Same as (f), but for the dataset acquired at a throughput of 80 samples per day.


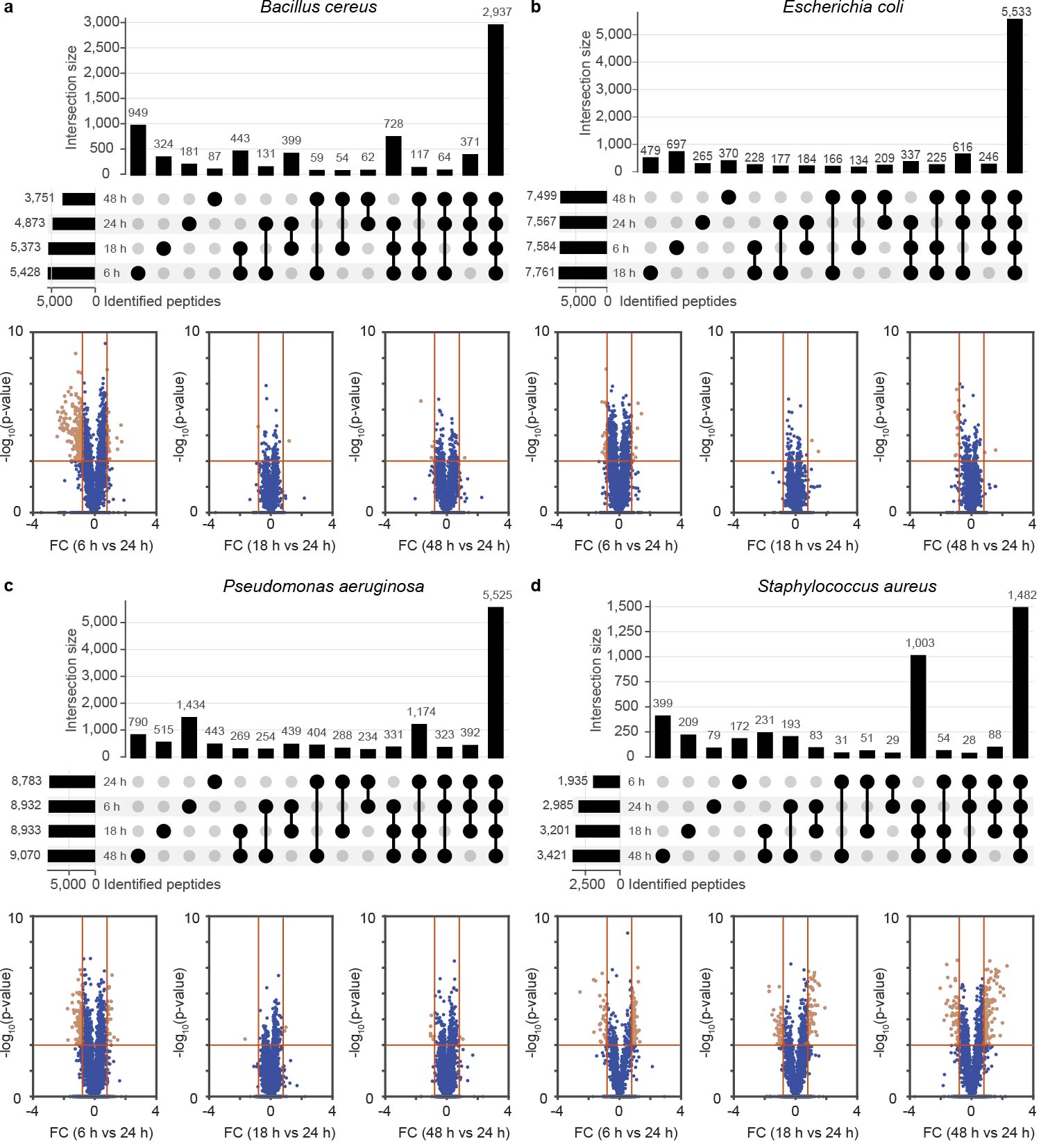


**Supplemental Figure 8: Bacterial sampling at variable cultivation time points leads to differential protein abundances**

1. *Bacillus cereus* was grown in triplicates for 6 h, 18 h, 24 h, and 48 h on Tryptic Soy Agar (TSA). A 1% MaxQuant search was performed to identify and quantify peptides. Upset plot (upper panel) depicts the intersection of tryptic peptides between conditions (no missed cleavages and modifications allowed). Bars on the left show how many peptides were identified at 1% FDR per condition (union of three replicates). Most peptides were found to be present in all four conditions. Volcano plots (lower panels) show quantitative differences between conditions. Samples taken after 6 h (left), 18 h (middle), and 48 h (right) were compared to samples taken at 24 h (‘control’). The adjusted –log10(p-value) is plotted on the y-axis, and the fold change (log2 LFQ intensity; FC) is on the x-axis. Cut-offs were set to an adjusted –log10(p-value) = 2 and LFQ intensity = ±2.
2. Same as (a), but *Escherichia coli*.
3. Same as (a), but *Pseudomonas aeruginosa*.
4. Same as (a), but *Staphylococcus aureus*.


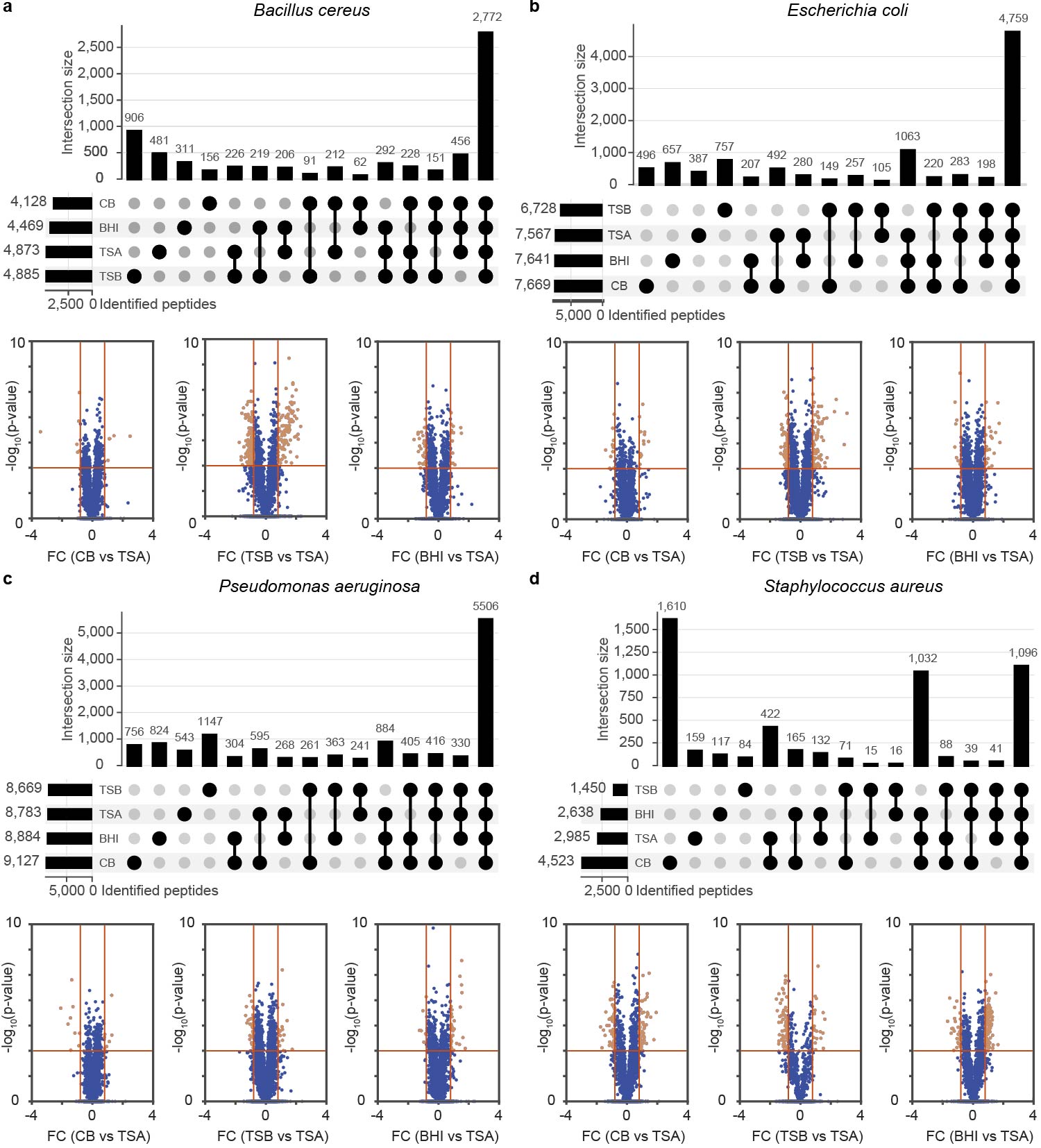


**Supplemental Figure 9: Bacterial sampling at variable cultivation conditions leads to differential protein abundances**

1. *Bacillus cereus* was grown on Tryptic Soy Agar (TSA), Columbia Blood Agar (CB), Brain-Heart Infusion Agar (BHI), and Tryptic soy Broth (TSB) for 24 h in triplicates. A 1% MaxQuant search was performed to identify and quantify peptides. Upset plot (upper panel) depicts the intersection of tryptic peptides (no missed cleavages and modifications allowed) between conditions. Bars on the left show how many peptides were identified at 1% FDR per condition (union of three replicates). Most peptides were found to be present in all four conditions. Volcano plots (lower panel) show quantitative differences between conditions. Samples incubated on CB (left), TSB (middle), and BHI (right) were compared to samples grown on TSA (control). The adjusted –log10(p-value) is plotted on the y-axis, and the fold change (log2 LFQ Intensity, FC) is plotted on the x-axis. Cut-offs were set to and adjusted –log10(p-value) = 2 and log2 LFQ intensity = ±2.
2. Same as (a), but *Escherichia coli*.
3. Same as (a), but *Pseudomonas aeruginosa*.
4. Same as (a), but *Staphylococcus aureus*.


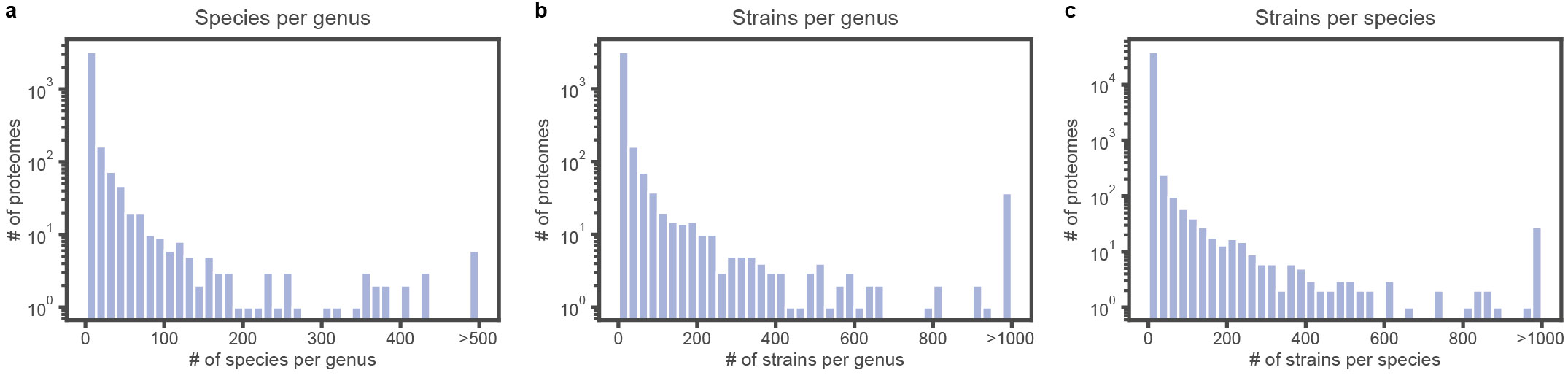


**Supplemental Figure 10: Strain-level reference databases’ composition**

1. Histogram displaying the number of proteomes with a certain number of species per genus.
2. Histogram displaying the number of proteomes with a certain number of strains per genus.
3. Histogram displaying the number of proteomes with a certain number of species per genus.

**
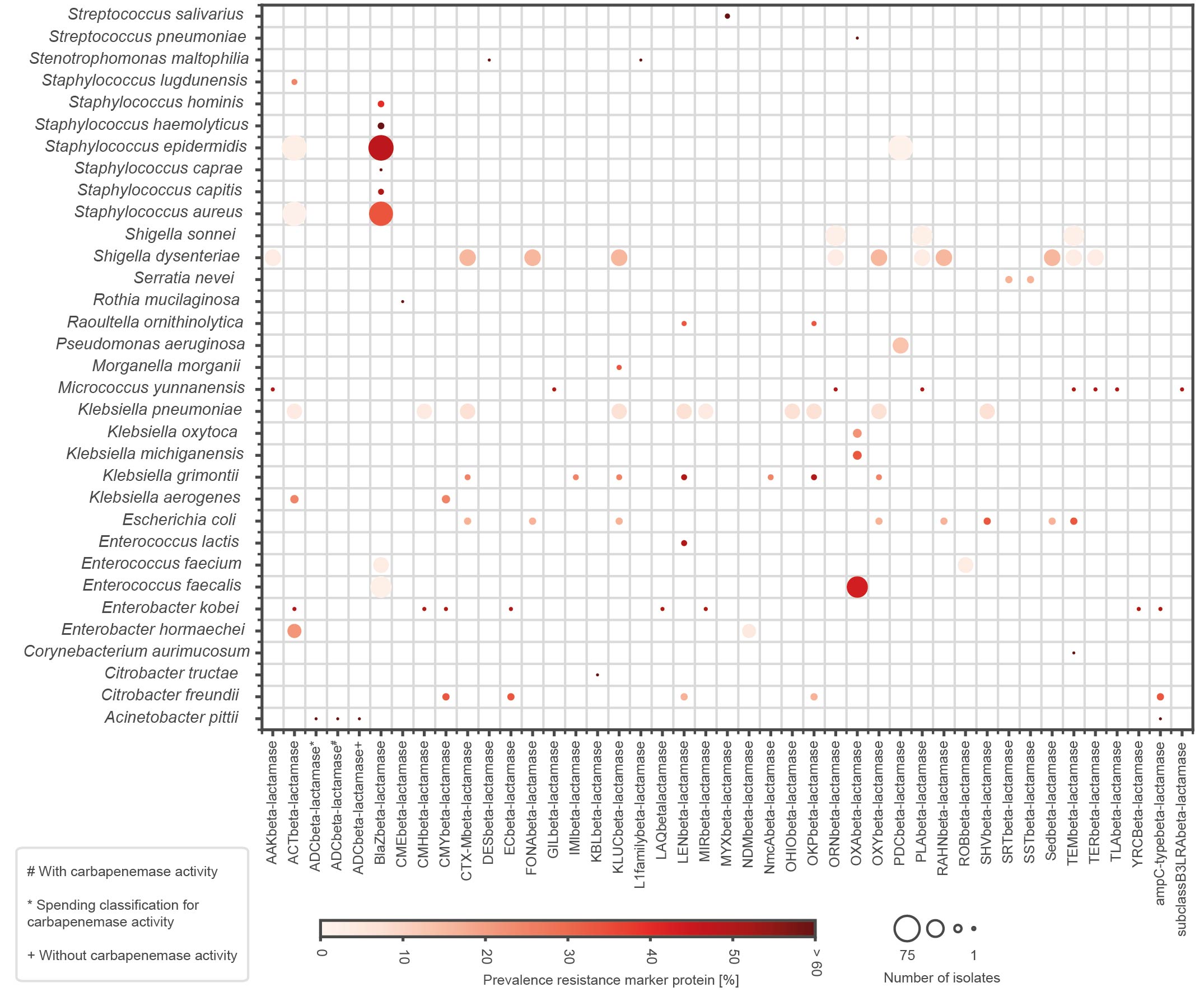
**

**Supplemental Figure 11: Beta-lactamase detection in clinical isolates**

Beta-lactamases were detected in many clinical isolates in a 1% FDR-controlled MaxQuant search. The circle size indicates the number of isolates with beta-lactamase detection per species, and the color coding indicates the prevalence of the respective beta-lactamase within the species. Only species for which a beta-lactamase was detected with at least two peptides are shown (iBAQ intensity > 0).

**Literature**

1. Claeys, T., et al., *lesSDRF is more: maximizing the value of proteomics data through streamlined metadata annotation.* Nat Commun, 2023. **14**(1): p. 6743.

2. Huang, Q., et al., *PaxDb 5.0: Curated Protein Quantification Data Suggests Adaptive Proteome Changes in Yeasts.* Mol Cell Proteomics, 2023. **22**(10): p. 100640.

3. Elias, J.E. and S.P. Gygi, *Target-decoy search strategy for mass spectrometry-based proteomics.* Methods Mol Biol, 2010. **604**: p. 55-71.
